# Enhancing soil health with *Bacillus velezensis* UFV 3918 boosts phosphorus and nutrient accumulation in sugarcane

**DOI:** 10.3389/fpls.2026.1805752

**Published:** 2026-04-23

**Authors:** Hariane Luiz Santos, Gustavo Ferreira da Silva, Adão de Siqueira Ferreira, Marcelo de Almeida Silva

**Affiliations:** 1 Laboratory of Ecophysiology Applied to Agriculture (LECA), Department of Crop Production, School of Agricultural Sciences, UNESP–Sao Paulo State University, Botucatu, Brazil; 2 Laboratory of Agricultural and Environmental Microbiology, Institute of Agricultural Sciences, Federal University of Uberlândia, Uberlândia, Brazil

**Keywords:** macronutrients, microbiological indicators, micronutrients, phosphate fertilization, phosphate-solubilizing bacteria, *Saccharum* spp.

## Abstract

Bacterial inoculation is a promising strategy for optimizing the use of phosphate fertilizers, although the ability of different strains to reduce phosphorus (P) inputs remains to be investigated. This study evaluated the effects of *Bacillus velezensis* UFV 3918 (Bv), alone or combined with monoammonium phosphate (MAP), on the bioavailability of P and other nutrients, as well as on the chemical and microbiological properties of soil cultivated with sugarcane. The trial was conducted in a completely randomized design with six treatments: absolute control (without MAP) (AC), commercial control (recommended MAP dose – 3/3 MAP) (CC), Bv, Bv+1/3 MAP, Bv+2/3 MAP, and Bv+3/3 MAP. The Bv and Bv+1/3 MAP treatments promoted average increases in soil P content (22.0%), root volume (9.9%), root diameter (6.7%), soil basal respiration (11.4%), and the activity of fluorescein diacetate (17.4%), urease (9.1%), and acid phosphatase (9.3%), compared with the CC. Additionally, Bv alone led to greater accumulation of P, K, Fe, B, and Cu in the shoot. Multivariate analysis indicated similar responses among Bv, Bv+1/3 MAP, and Bv+2/3 MAP. Shoot P accumulation was positively correlated with microbial and chemical soil attributes, particularly soil enzyme activities, P and Ca content, and Fe and Mn accumulation. In contrast, the negative correlation between microbial biomass and root development suggests that enhanced soil biological activity may reduce the need for extensive root growth. Thus, *B. velezensis* UFV 3918 shows potential to reduce MAP dependence while promoting efficient plant nutrition and healthier soils.

## 1 Introduction

Sugarcane is a vital crop for renewable energy production worldwide, covering approximately 24.3 million hectares globally (OECD and Food and Agriculture Organization of the United Nations, 2022). Brazil is the largest sugarcane producer, producing 713.2 million tons during the 2023/2024 harvest on 8.33 million hectares of cultivated land (Conab – Companhia Nacional de Abastecimento, 2024).

Synthetic chemical fertilizers have been used to optimize crop growth and productivity. However, plant nutrient uptake and crop productivity have not significantly improved (Seleiman et al., 2020; Yokamo et al., 2023). Mineral fertilizers generally exhibit low efficiency in agricultural systems (Guo et al., 2018). Phosphorus (P) use efficiency, for instance, is estimated to range from 18% to 20%, which has remained constant over the past decades (Preetha and Balakrishnan, 2017; Khan et al., 2023). This inefficiency indicates that more than half of the phosphate applied as fertilizer is lost due to immobilization (Solangi et al., 2023).

Soil phosphorus availability is affected by fixation, primarily through binding to iron (Fe) and aluminum (Al) oxides, a process that is intensified in the acidic soils typical of tropical regions. This reduces plant P uptake (Asomaning, 2020; Hanyabui et al., 2020; Fahad et al., 2022). Consequently, the low efficiency of P use necessitates the intensive application of synthetic phosphate fertilizers to enhance agricultural productivity (Guo et al., 2018). In Brazil, these challenges are compounded by the fact that approximately 75% of mineral phosphate fertilizers used in agriculture are imported (Colussi et al., 2022). This dependence raises global concerns about the costs and energy involved in phosphate mining and transportation. Such concerns highlight the importance of minimizing waste and promoting nutrient cycling through biofertilizers and sustainable practices (Sharma et al., 2013; Meng et al., 2019; Benites et al., 2023).

Soils used for sugarcane cultivation often have a high P fixation capacity due to frequent harvesting and challenges in incorporating fertilizers into ratoon cane. As a result, large quantities of soluble phosphate fertilizers are required to maintain adequate P levels for satisfactory crop yields (Roy et al., 2016; Estrada-Bonilla et al., 2021). However, in the long term, the intensive use of these fertilizers can lead to environmental risks, including soil degradation (Seleiman et al., 2020; Pahalvi et al., 2021; Peñuelas et al., 2023).

A sustainable approach to increasing P availability (Billah et al., 2019; Shukla, 2019; Rosa et al., 2020, 2022; Li et al., 2023) and improving P use efficiency in the soil–plant system (Kumar et al., 2017; Elhaissoufi et al., 2022) involves the use of phosphate-solubilizing bacteria (PSBs). These microorganisms can solubilize and mineralize inorganic and organic P sources (Kalayu, 2019; Rawat et al., 2021). Key mechanisms include the production of organic acids, siderophores, protons, and CO_2_ (Sharma et al., 2013; Kishore et al., 2015; Kumar and Rai, 2015; Ferreira et al., 2019; Wang and Lambers, 2020a, b) as well as the action of enzymes like phosphatases, phytases, and phosphonatases (Behera et al., 2013; Sharma et al., 2013; Kumar and Shastri, 2017; Teng et al., 2019). Bacteria of the genus *Bacillus* are particularly abundant in the rhizosphere and show great promise as phosphate solubilizers (Saeid et al., 2018; Afzal et al., 2022; Mosela et al., 2022). Using these microorganisms promotes sustainable agriculture by enhancing soil health, which benefits ecosystems by maintaining water quality, improving plant productivity, and regulating nutrient cycling (Tahat et al., 2020; Santos et al., 2022).

Beyond increasing P availability, PSBs can also benefit crops by stimulating root growth (Luo et al., 2024). Rhizosphere microorganisms, in association with roots, modulate the root system through various mechanisms to enhance nutrient uptake, even under limiting conditions (Hussain et al., 2019; Emami et al., 2019; Elhaissoufi et al., 2020; Bargaz et al., 2021).

The activity of microbial communities in soil can be assessed to understand the mechanisms by which bacteria solubilize P. These assessments can be carried out using microbiological indicators, enzyme activities linked to nutrient biogeochemical cycles, and enzyme activities in bacterial intracellular metabolism (Sobucki et al., 2021). *Bacillus velezensis* UFV 3918 has been shown to increase soil basal respiration, microbial biomass carbon, fluorescein diacetate hydrolysis, and phosphatase activity, all of which are associated with soil health in sugarcane fields (Santos et al., 2022).

Although previous studies have demonstrated the potential of phosphate-solubilizing bacteria to improve P availability, limited information remains on the effects of *Bacillus velezensis* inoculation combined with mineral phosphate fertilization on soil microbial activity and plant nutrient accumulation in sugarcane. Understanding these interactions is essential for improving P use efficiency and optimizing fertilization strategies.

Therefore, the objective of this research was to evaluate the effects of *B. velezensis* UFV 3918 combined or not with doses of monoammonium phosphate (MAP) on soil microbiological properties, root growth, nutrient bioavailability, and nutrient accumulation in sugarcane shoots 180 days after planting, providing an integrated assessment of soil–plant interactions. We hypothesize that inoculating sugarcane buds with *B. velezensis* UFV 3918 improves soil microbiological activity, enhances P availability, and consequently favors nutrient accumulation in shoots, even with reduced MAP application.

## 2 Materials and methods

### 2.1 Cultivation conditions and plant material

The experiment was conducted from November 2021 to May 2022 in a greenhouse at the Department of Crop Production, School of Agricultural Sciences, UNESP, in Botucatu, SP, Brazil (22°51’01”S, 48°25’55”W, 786 m asl).

The greenhouse structure was built with galvanized steel, featuring an arched roof. The ceiling height ranged from 3.6 m at the sides to 4.8 m at the center, covering an area of 36 m². The sides were fitted with a 2 mm polyethylene anti-aphid screen that provided 14% shading, while the roof was constructed from 150 μm transparent plastic.

To control soil pathogens, the solarization technique was used as a disinfection method (Patrício et al., 2005). This approach aimed to minimize the impact of microorganisms unrelated to the strain under study on plant sprouting, growth, and phosphate solubilization. The soil used in the experiment was classified as dystrophic Red Latosol (Santos et al., 2018). Granulometric analysis indicated a composition of 68.2% sand, 25.7% clay, and 6.1% silt, categorizing it as a medium-textured soil.

The sugarcane variety RB966928 was chosen for this study due to its prevalence in Brazil’s sugarcane-producing regions. It represents 17.7% of the cultivated area in São Paulo state (Braga Junior et al., 2021). The plants were irrigated to 100% pot capacity using a drip irrigation system (Netafim, PCJ-CNL 4 L/h, Ribeirão Preto, SP, Brazil), with flow rates regulated by valves.

### 2.2 Description, production, and origin of the product

The commercial product used in this study is a liquid formulation (density 1.03 g mL^–1^) containing the active ingredient *Bacillus velezensis* UFV 3918 at a concentration of 1.0 × 10^8^ colony-forming units (CFU) mL^–1^. The formulation consists of endospores, metabolites released during bacterial growth, and a stabilizing agent and does not contain other microorganisms or chemical additives. The recommended application rate for sugarcane is 2 L ha^–1^. The product, formulated based on the UFV 3918 strain, was supplied by Vittia (São Joaquim da Barra, SP, Brazil). A commercial formulation was used to simulate practical agricultural conditions while maintaining experimental rigor.

### 2.3 Treatments and experimental design

The experiment was designed as a completely randomized trial, consisting of six treatments [Absolute Control (AC) – without MAP; Commercial Control (CC) – full recommended dose of MAP (3/3 MAP); *Bacillus velezensis* UFV 3918 (Bv); Bv+1/3 MAP; Bv+2/3 MAP; and Bv+3/3 MAP], with four replicates each.

Before treatment application, the soil had a pH (CaCl_2_) of 6.0, an organic matter content of 40.1 g dm^–3^, and low exchangeable acidity (Al^3+^ = 0.7 mmol_c_ dm^–3^), with a potential acidity (H + Al) of 20.8 mmol_c_ dm^–3^. The exchangeable K, Ca, and Mg concentrations were 0.6, 75.2, and 26.4 mmol_c_ dm^–3^, respectively. The sum of bases (SB) was 102.2 mmol_c_ dm^–3^, and the cation exchange capacity (CEC) reached 123.0 mmol_c_ dm^–3^, resulting in a base saturation (V%) of 83.0%. Available phosphorus (P_resin_) was 61.8 mg dm^–3^, and sulfur (S) was 34.0 mg dm^–3^. Micronutrient concentrations were: Cu = 0.3, Fe = 22.7, Mn = 0.7, Zn = 1.2, and B = 0.2 mg dm^–3^.

Each experimental unit comprised 50 L pots filled with 45 dm³ of soil. The soil was chemically corrected based on chemical analysis (Vitti et al., 2015), with fertilizers incorporated at planting. Different fertilization strategies were adopted using varying doses of monoammonium phosphate (MAP, containing 60% soluble P in neutral ammonium citrate and 12% N), combined with fixed rates of KCl and urea. The recommended MAP dose treatment received 125 kg ha^–1^ of MAP (2.8 g pot^–1^), 250 kg ha^–1^ of KCl (5.635 g pot^–1^), and 36.12 kg ha^–1^ of urea (0.813 g pot^–1^). The 2/3 MAP treatment received 83.33 kg ha^–1^ of MAP (1.878 g pot^–1^), 46.3 kg ha^–1^ of urea (1.04 g pot^–1^), and the same KCl dose. The 1/3 MAP treatment received 41.7 kg ha^–1^ of MAP (0.939 g pot^–1^), 56.47 kg ha^–1^ of urea (1.272 g pot^–1^), and 250 kg ha^–1^ of KCl. The treatment without MAP received only urea (66.6 kg ha^–1^; 1.5 g pot^–1^) and KCl (250 kg ha^–1^; 5.635 g pot^–1^), without phosphorus addition at planting. Additionally, urea was top-dressed at 1.5 g pot^–1^ (equivalent to 30 kg N ha^–1^) before stalk formation, as recommended for medium-textured soils (Santos et al., 2018).

Planting occurred on November 12, 2021, and fertilizers were incorporated into the soil. Bacterial inoculation involved applying 2 mL of the commercial product containing *B. velezensis* UFV 3918 (10^8^ CFU mL^–1^) diluted in 75 mL of pH 7.0 water per pot. For treatments with bacterial inoculation, each bud received 15.4 mL of this solution, while buds in other treatments were irrigated with the same volume of water. The buds were planted at a depth of 2 cm. Plants were harvested on May 2022, corresponding to 180 days after planting (DAP).

### 2.4 Biomass production

At 180 DAP, the plants were harvested and partitioned to obtain leaf biomass (LB), leaf sheath biomass (LSB), stalk biomass (SB), and root biomass (RB). The plant parts were kept in a forced-air circulation oven at 65 °C until their mass was constant, and then weighed on a 0.01 g precision scale (Balmak, ELC−6/15/30, Santa Bárbara d’Oeste, SP, Brazil).

For LB, all leaves produced by the plant (dry and green leaves) were considered; for LSB, all sheaths produced by the plant (dry and green sheaths) were considered; for SB, all tillers in the pot were considered (from the soil base to the apical meristem), and for RB, the root system of the clump was considered.

### 2.5 Sampling and chemical analysis of plant tissue

At harvest, median sections of the +1 leaves were collected. The +1 leaf is the first leaf with a fully expanded ligule, commonly called the top visible dewlap (TVD) leaf (van Raij et al., 1997). The leaf midrib was excluded from the sampling. Additionally, leaf sheaths of the +1 leaves and median portions of the main stalks were collected. The sampled plant material was dried in a forced-air oven at 60 °C until constant weight was achieved, then ground using a Wiley-type mill.

Nitrogen (N) in leaf tissue was extracted by sulfuric acid digestion and quantified by the Kjeldahl method. The extraction of K, Ca, Mg, S, P, Fe, Zn, Mn, B, and Cu was performed using nitroperchloric digestion, following AOAC (2016) guidelines. K, Ca, Mg, Fe, Zn, Mn, and Cu concentrations were determined by AAS, while S, P, and B concentrations were analyzed by colorimetric methods.

The accumulation of nutrients in the shoot was calculated as follows (Equation 1):

(1) Ac=SB ×NC

Where Ac represents nutrient accumulation (g or mg plant^−1^), SB is the shoot biomass (g), and NC is the nutrient concentration (g or mg kg^−1^).

### 2.6 Root variables

Roots were carefully washed under running water at harvest, and the root system (RS) was sampled. The RS was chopped and homogenized, and a uniform sample representing the RS’s superficial, central, lateral, and terminal portions was collected. The remaining RS was placed in paper bags and dried in a forced-air oven at 65 °C until constant weight was achieved. Subsequently, the dried roots were weighed using a precision scale (Balmak, ELC-6/15/30, Santa Barbara d’Oeste, SP, Brazil) to determine root biomass (RB).

Root samples were preserved in 70% (v/v) ethanol vials and stored at refrigeration temperature. These samples were later scanned at 250 dpi, and the resulting images were analyzed with WinRhizo. The analysis provided measurements of root volume (RV), mean root diameter (RD), root surface area (RSA), and root surface projection (RSP) (Tennant, 1975). The scanned samples were then placed in paper bags and oven-dried at 65 °C for 48 hours to determine their dry matter mass.

### 2.7 Soil sampling and chemical analysis

Soil samples were collected at harvest from 0–0.15 m depth. The collected soil was dried in a forced-air oven at 40 °C for 96 hours and sieved through a 2 mm mesh.

Organic matter (OM) content was determined following the Walkley and Black (1934) method. Concentrations of potassium (K), calcium (Ca), magnesium (Mg), sulfur (S), phosphorus (P), iron (Fe), zinc (Zn), manganese (Mn), and copper (Cu) were analyzed according to the protocol described by van Raij et al. (2001).

Macronutrients were extracted using an ion exchange resin. K, Ca, and Mg concentrations were measured via atomic absorption spectrophotometry (AAS), while S and P were quantified through colorimetric analysis. Micronutrients were extracted using a solution containing diethylenetriaminepentaacetic acid (DTPA) (0.005 M, pH 7.3), triethanolamine (TEA) (0.1 M), and CaCl_2_ (0.01 M). The resulting micronutrient concentrations were also determined by AAS (van Raij et al., 2001).

### 2.8 Soil microbiological indicators

Soil samples for microbiological analyses were collected at 0–0.10 m immediately after sugarcane harvest and stored at –10 °C until analysis.

Basal soil respiration (BSR) was assessed by measuring C–CO_2_ release during the static incubation of 50 g of soil in airtight glass jars maintained at 25 °C in a biochemical oxygen demand (BOD) incubator (Silva et al., 2007). The C–CO_2_ released was quantified at 3, 7, 14, and 21 days after incubation by titration with HCl after adding Ba(OH)_2_ to precipitate carbonates. The cumulative C–CO_2_ release over 21 days was expressed in µg C–CO_2_ g^−1^ dry soil.

Microbial biomass carbon (MBC) and nitrogen (MBN) were determined using the irradiation-extraction method (Vance et al., 1987; Ferreira et al., 1999). For MBC, soil organic carbon was extracted using K_2_SO_4_ (0.5 mol L^−1^, pH 6.8), and the carbon concentration difference between irradiated and non-irradiated samples was multiplied by a conversion factor (K_C_ = 0.33) to estimate MBC. Results were expressed as µg C g^−1^ soil (Ferreira et al., 1999).

Nitrogen extraction for MBN involved shaking soil with K_2_SO_4_ solution, followed by digestion with H_2_SO_4_ and a catalyst mixture (K_2_SO_4_, CuSO_4_, and selenium powder). The digested samples were distilled, and the distillate was titrated with H_2_SO_4_. The difference in nitrogen concentrations between irradiated and non-irradiated samples was multiplied by a factor (K_N_ = 0.54) to estimate MBN, expressed in µg N g^−1^ soil (Ferreira et al., 1999).

Catalytic enzyme activity, both intra- and extracellular, was evaluated by fluorescein diacetate (FDA) hydrolysis, following the method proposed by Green et al. (2006). Soil samples (1 g) were mixed with 5 mL of potassium phosphate buffer (pH 7.6) and 0.2 mL of FDA substrate in Falcon tubes. After incubation at 30 °C for 1 h, fluorescein was extracted with a chloroform-methanol solution (2:1). A 2 mL aliquot of the supernatant was centrifuged at 5000×g for 10 min, and sodium fluorescein quantification was performed using a standard curve. Absorbance was measured at 490 nm using a spectrophotometer (Thermo Fisher Scientific, BioMate™ 3, Waltham, MA, USA). Results were expressed as µg FDA g^−1^ dry soil h^−1^.

Dehydrogenase activity was assessed following the methodology of Mersi and Schinner (1991). One gram of soil was incubated with tris(hydroxymethyl)-aminomethane buffer (1 mol L^–1^, pH 7.5) and an INT (2-p-iodophenyl-3-p-nitrophenyl-5-phenyltetrazolium bromide) substrate (4.4 mmol L^–1^) at 40 °C for 5 h. The enzymatic reaction product, iodo-nitrophenylformazan (INTF), was extracted with an ethanol-dimethylformamide solution (1:1). After incubation, the mixture was centrifuged at 5000×g for 10 min, and the absorbance of the supernatant was measured at 490 nm. Results were expressed as μg INTF g^−1^ soil h^−1^.

Urease activity was determined using the method described by Kandeler and Gerber (1988). Soil samples of 1 g were incubated with citrate buffer (pH 6.7) and a 10% urea solution at 37 °C for 2 h. The ammonium nitrogen (N–NH_4_) released during the reaction was quantified at 600 nm using a spectrophotometer (Thermo Fisher Scientific, BioMate™ 3, Waltham, MA, USA) with the commercial Urea 500^®^ kit (Doles Inc., Goiânia, GO, Brazil). Results were expressed in µg N-NH4 g^–1^ of dry soil h^–1^.

The activity of β-glucosidase was evaluated following the protocol of Eivazi and Tabatabai (1988). Soil samples of 1 g were incubated with the substrate PNPG (*p*-nitrofenil-β-D-glucopiranósido) (4.2 mmol L^–1^) in a modified universal buffer (MUB, pH 6.0) at 37 °C for 1 h. Afterward, CaCl_2_ (1_mol_ L^-1^) and NaOH (1 mol L^–1^) were added to terminate the reaction. The supernatant was centrifuged, and the product, p-nitrophenyl (PNP), was quantified spectrophotometrically at 410 nm. Results were presented as micrograms of PNP per gram of dry soil per hour. A 2 mL aliquot of the supernatant was centrifuged at 5,000×g for 10 min, and the product, p-nitrophenol (PNP), was quantified at 410 nm using a spectrophotometer (Thermo Fisher Scientific, BioMate™ 3, Waltham, MA, USA). Results were expressed in µg PNP g^−1^ dry soil h^−1^.

Arylsulfatase activity was measured using a similar approach to β-glucosidase activity, with the substrate replaced by p-nitrophenyl sulfate (0.05 mol L^–1^) in a sodium acetate buffer (pH 5.8). The enzymatic reaction product was expressed in µg PNP g^−1^ dry soil h^−1^.

Acid phosphatase activity was determined by measuring the release of PNP from the substrate p-nitrophenyl phosphate (2.4 mmol L^–1^) in MUB (pH 6.0), as described by Tabatabai and Bremner (1969). The soil samples were incubated at 37 °C for 1 h, and then CaCl_2_ (1 mol L^–1^) and NaOH (1 mol L^–1^) were added. A 2 mL aliquot of the supernatant was centrifuged at 5000×g for 10 min. The released PNP was measured in a spectrophotometer (Thermo Fisher Scientific, BioMate™ 3, Waltham, MA, USA) at 405 nm. Results were expressed in µg PNP g^−1^ dry soil h^−1^.

### 2.9 Statistical analysis

The data were subjected to normality (Shapiro-Wilk) and homoscedasticity (Levene) tests and, after meeting the assumptions, were subjected to analysis of variance (ANOVA) with the F test, with subsequent comparison of means by the Tukey test (*p ≤* 0.05) using the AgroEstat statistical software (AgroEstat, version 2015, Jaboticabal, SP, Brazil). Additionally, the data were subjected to regression adjustment for the MAP doses associated with *B. velezensis* (Bv, Bv+1/3 MAP, Bv+2/3 MAP, and Bv+3/3 MAP) using Minitab statistical software (Minitab^®^, version 19, State College, PA, USA). The figures were generated using SigmaPlot software (SigmaPlot^®^, version 14.0, Systat Software, CA, USA).

To identify patterns and differentiate treatments, Partial Least Squares Discriminant Analysis (PLS-DA), a supervised dimensionality-reduction method that maximizes group separation, was applied using MetaboAnalyst 6.0 (Pang et al., 2024). Initially, the data were transformed using base-10 logarithms and normalized to minimize unwanted variability and improve comparability across samples.

The importance of variables in discriminating among treatments was assessed using VIP (Variable Importance in Projection) scores; variables with VIP scores greater than 1.0 were considered relevant. The 20 most important variables were analyzed to interpret the results.

Additionally, a heatmap was generated to visualize the distribution of variables among treatments. Statistical evaluation included one-way ANOVA and t-tests, while hierarchical clustering was performed using Ward’s method and the Euclidean distance measure. Pearson’s linear correlation coefficient (*p* < 0.05) was used to evaluate the relationship between variables.

## 3 Results

### 3.1 Soil microbiological indicators

**Figure 1:**
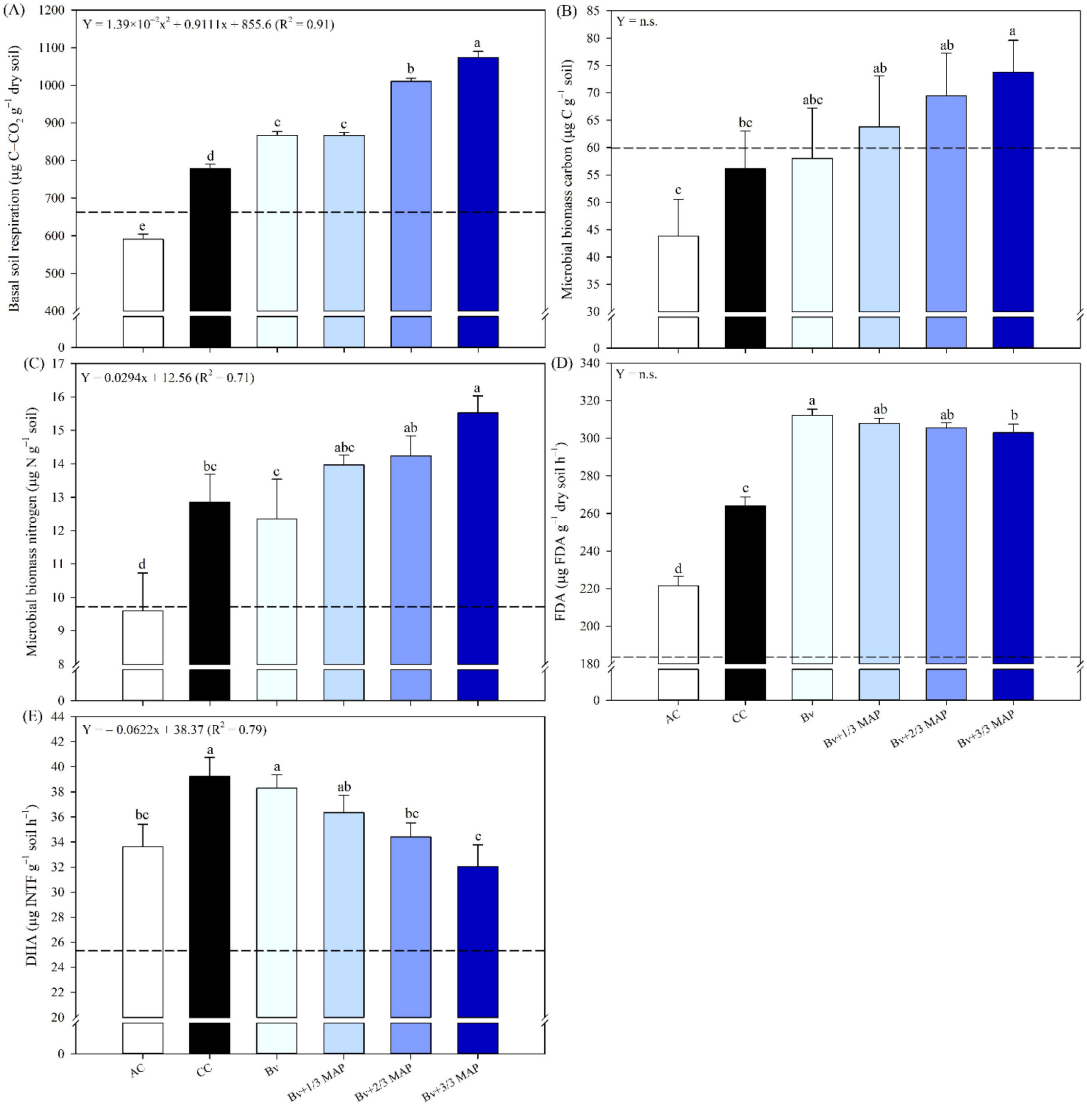
Soil basal respiration accumulated in 21 days **(A)**, microbial biomass carbon and nitrogen **(B, C)**, fluorescein diacetate (FDA) hydrolysis **(D)**, and dehydrogenase activity (DHA) **(E)** in soil cultivated with sugarcane, under treatments with and without inoculation of *B. velezensis* UFV 3918 (Bv) and mono ammonium phosphate (MAP) doses, 180 days after planting. Means followed by the same letter do not differ at a 5% probability level, according to Tukey’s test. Error bars express the standard deviation of the mean (n = 4), and dashed horizontal lines represent the initial soil condition before cultivation. Regression equations and R^2^ refer to the association between *B. velezensis* UFV 3918 and MAP doses at the 5% significance level (*). AC, absolute control; CC, commercial control.

Considering the accumulated BSR over 21 days, the highest values were observed in the treatments inoculated with *B. velezensis* (**Figure 1A**). AC was the only treatment that did not exceed the BSR assessed before cultivation (662.39 µg C–CO_2_ g^–1^ dry soil). Bv and Bv+1/3 MAP, Bv+2/3 MAP, and Bv+3/3 MAP provided increases of 11.4%, 29.9%, and 38.1%, respectively, compared with CC. The rise in RBS (0.91*) was directly proportional to the increase in MAP doses associated with *B. velezensis*.

Regarding CMB, there was no difference between the inoculated treatments for this variable, and only Bv+3/3 MAP differed from CC, representing a 31.5% increase (**Figure 1B**). Only Bv+1/3, 2/3, and 3/3 MAP exceeded the CMB observed in the soil before cultivation (69.92 µg C g^–1^ soil).

Bv, Bv+1/3 MAP, and Bv+2/3 MAP were similar to CC for NMB, while Bv+3/3 MAP provided a 20.8% increase in NMB compared with CC (**Figure 1C**). Except for AC, the other treatments resulted in NMB higher than that observed in the soil before cultivation (9.72 µg N g^–1^ soil). Considering Bv + MAP doses (0.71*), as MAP doses increased, there was a linear increase in NMB.

Regarding the FDA, the highest values were observed in the treatments inoculated with *B. velezensis*. Among these, Bv promoted the highest FDA, representing an 18.2% increase compared with CC (**Figure 1D**). All treatments exceeded the FDA observed in the initial soil (183.59 µg FDA g^–1^ dry soil h–1), with the greatest increase observed in Bv (70%).

In contrast to the BSR, increasing P doses with *B. velezensis* reduced DHA activity. Bv and CC showed the highest DHA activities, with an average increase of 15.3% compared with AC (**Figure 1E**). All treatments exceeded the enzyme activity observed in the initial soil (25.32 µg INTF g^–1^ soil h^–1^). Considering Bv + MAP doses (0.79*), 0% MAP was the optimal dose, as it provided similar enzyme activity to that found in CC (100% MAP).

**Figure 2:**
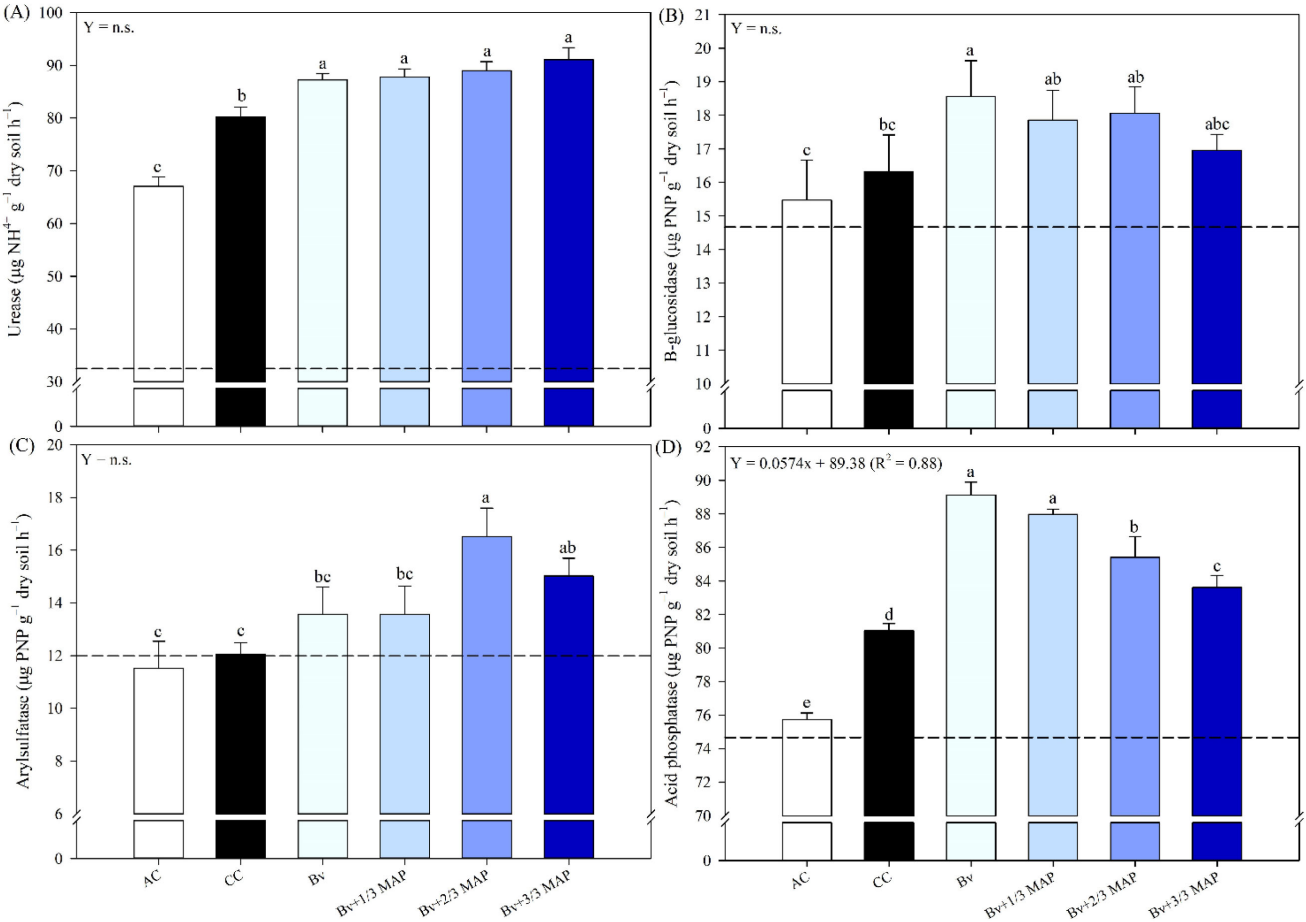
Activity of the enzymes urease **(A)**, β-glucosidase **(B)**, arylsulfatase **(C)**, and acid phosphatase **(D)** in soil cultivated with sugarcane, under treatments with and without inoculation of B. velezensis UFV 3918 (Bv) and doses of mono ammonium phosphate (MAP), 180 days after planting. Means followed by the same letter do not differ at a 5% probability level, according to Tukey’s test. Error bars express the standard deviation of the mean (n = 4), and dashed horizontal lines represent the initial soil condition before cultivation. Regression equations and R^2^ refer to the association between *B. velezensis* UFV 3918 and MAP doses at the 5% significance level (*). AC, absolute control; CC, commercial control.

The highest urease activities were observed in the treatments inoculated with *B. velezensis*, which were similar to each other and provided average increases of 10.7% and 32.4% compared with CC and AC, respectively (**Figure 2A**). All treatments surpassed the urease activity observed in the initial soil (32.48 µg NH_4_^+^ g^–1^ dry soil h^–1^). Still, the most significant increases were observed in Bv+2/3 MAP and Bv+3/3 MAP (174% and 180.3%, respectively).

As the MAP doses combined with *B. velezensis* increased, a trend toward decreased β-glucosidase activity was observed; however, no dose-response relationship was established. Bv+1/3 MAP, Bv+2/3 MAP, and Bv+3/3 MAP exhibited enzymatic activity comparable to the CC (**Figure 2B**). Although Bv did not differ significantly from the other inoculated treatments, it led to a 13.8% increase in β-glucosidase activity compared with CC. All treatments exceeded the enzymatic activity measured in the initial soil (14.67 µg PNF g^–1^ dry soil h^–1^).

Regarding arylsulfatase activity, Bv and Bv+1/3 MAP showed values similar to those of CC. At the same time, Bv+2/3 MAP and Bv+3/3 MAP showed the highest activities, with increases of 37.0% and 24.6%, respectively, compared with CC (**Figure 2C**). Except for the AC, all treatments surpassed the arylsulfatase activity observed in the pre-planting soil (11.99 µg PNF g^–1^ dry soil h^–1^), with average increases of 13.2% (Bv and Bv+1/3 MAP), 37.7% (Bv+2/3 MAP), and 25.3% (Bv+3/3 MAP).

The highest acid phosphatase (AP) activities were found in inoculated treatments (**Figure 2D**). Bv and Bv+1/3 MAP promoted increases of 10% and 8.6%, respectively, in AP activity compared with CC. Considering Bv+ MAP doses (0.88*), there was a trend of linear reduction in enzyme activity as MAP doses increased. Nevertheless, all treatments exceeded the AP activity measured in the initial soil (74.66 µg PNF g^–1^ dry soil h^–1^) (**Figure 2D**).

### 3.2 Root variables

The treatments without phosphate fertilization (AC and Bv) performed best for root-related variables (**Table 1**). The highest RV was observed in Bv, representing increases of 30.5%, 18.7%, 17.2%, and 32.8% compared with CC, Bv+1/3 MAP, Bv+2/3 MAP, and Bv+3/3 MAP, respectively (**Table 1**). Increasing MAP doses, in combination with Bv, reduced RV, with a negative linear trend (0.85*). There were no significant differences among the inoculated plants for RD, although Bv+1/3 MAP showed a RD value 6.7% higher than that of CC (**Table 1**). No significant regression was observed for RD considering Bv + MAP doses.

**Table 1:** Root volume (RV), average root diameter (RD), root surface area (RSA), root surface projection (RSP), and root biomass (RB) of sugarcane 180 days after planting, under treatments with and without *B. velezensis* UFV 3918 (Bv) inoculation and monoammonium phosphate (MAP) doses.

Treatments	Volume (cm^3^)	Diameter (mm)	Surface area (m^2^)	Surface projection (m^2^)	Root biomass (g)
AC	358.10 a	0.354 b	4.05 a	1.29 a	147.86 a
CC	276.23 c	0.360 b	3.28 b	1.05 b	127.06 b
Bv	360.53 a	0.363 ab	4.16 a	1.33 a	155.33 a
Bv+1/3 MAP	303.60 b	0.384 a	3.10 b	0.99 b	128.30 b
Bv+2/3 MAP	307.62 b	0.366 ab	3.30 b	1.05 b	126.36 bc
Bv+3/3 MAP	271.55 c	0.360 ab	3.14 b	1.00 b	117.54 c
C.V. (%)	6.89	4.91	5.52	7.72	8.12
Regression	Y = 4.759.10^–3^ x^2^ – 1.266x + 355.4 (R^2^ = 0.85)	Y = n.s.	Y = 2.01×10^-4^x^2^ – 0.029x + 4.071 (R^2^ = 0.77)	Y = n.s.	Y = 4.130×10^–3^x^2^ – 0.7529x + 153.7 (R^2^ = 0.88)

C.V., coefficient of variation. Means followed by the same letter do not differ using the Tukey test at 5% probability. The regression equations and R^2^ refer to the association between *B. velezensis* UFV 3918 and MAP doses at a significance level of 5% (*). n.s., not significant; AC, absolute control; CC, commercial control.

Bv promoted increases of 26.8% and 26.7% in RSA and RSP, respectively, compared with CC (**Table 1**). As MAP doses increased (0.77*), Bv + MAP doses reduced RSA. And there was no adjustment of Bv + MAP doses for RSP.

Regarding RB, the highest values were observed in the AC and Bv treatments. Bv promoted RB values by 22.2%, 21.1%, 25.3%, and 32.1% over those recorded for CC, Bv+1/3 MAP, Bv+2/3 MAP, and Bv+3/3 MAP, respectively (**Table 1**). Similar to the other root traits, RB decreased with increasing MAP doses, showing a negative linear trend (0.88*). These results indicate that the most favorable root system development occurred without phosphate fertilization.

### 3.3 Soil chemistry

No differences were observed among treatments for soil pH, potential acidity (H^+^ + Al^3+^), and base saturation (V%) (**Table 2**). The lowest Al^3+^ concentrations were found under Bv and Bv+2/3 MAP. For the sum of bases and cation exchange capacity (CEC), the highest values were observed under Bv and CC, representing average increases of 18.7% and 15.4%, respectively, compared with AC. No regression trends were detected for pH, H^+^ + Al^3+^, Al^3+^, SB, CEC, and V% as a function of Bv + MAP doses (**Table 2**).

**Table 2:** Final chemical parameters of the sugarcane cultivation soil under the association of *B. velezensis* (Bv) and monoammonium phosphate (MAP) doses.

Treatments	pH	H + Al^3+ 1^	Al^3+^	SB^2^	CEC^3^	V%^4^
AC	6.09 a	11.67 a	0.75 a	64.47 c	76.15 c	84.65 a
CC	6.08 a	12.04 a	0.70 a	77.71 a	89.75 a	86.58 a
Bv	6.15 a	11.58 a	0.45 b	77.91 a	89.49 a	87.06 a
Bv+1/3 MAP	6.16 a	12.48 a	0.90 a	71.91 b	84.40 b	85.22 a
Bv+2/3 MAP	6.15 a	12.83 a	0.32 b	74.87 ab	87.70 ab	85.36 a
Bv+3/3 MAP	6.14 a	12.38 a	0.72 a	72.61 b	84.99 b	85.42 a
C.V. (%)	2.48	8.38	10.03	5.00	4.37	3.44

Means followed by the same letter do not differ from each other by the Tukey test at 5% probability. ^1^H + Al^3+^ (Potential acidity). ^2^SB (sum of bases). ^3^CEC (cation exchange capacity). ^4^V% (base saturation). AC, absolute control; CC, commercial control.

**Figure 3:**
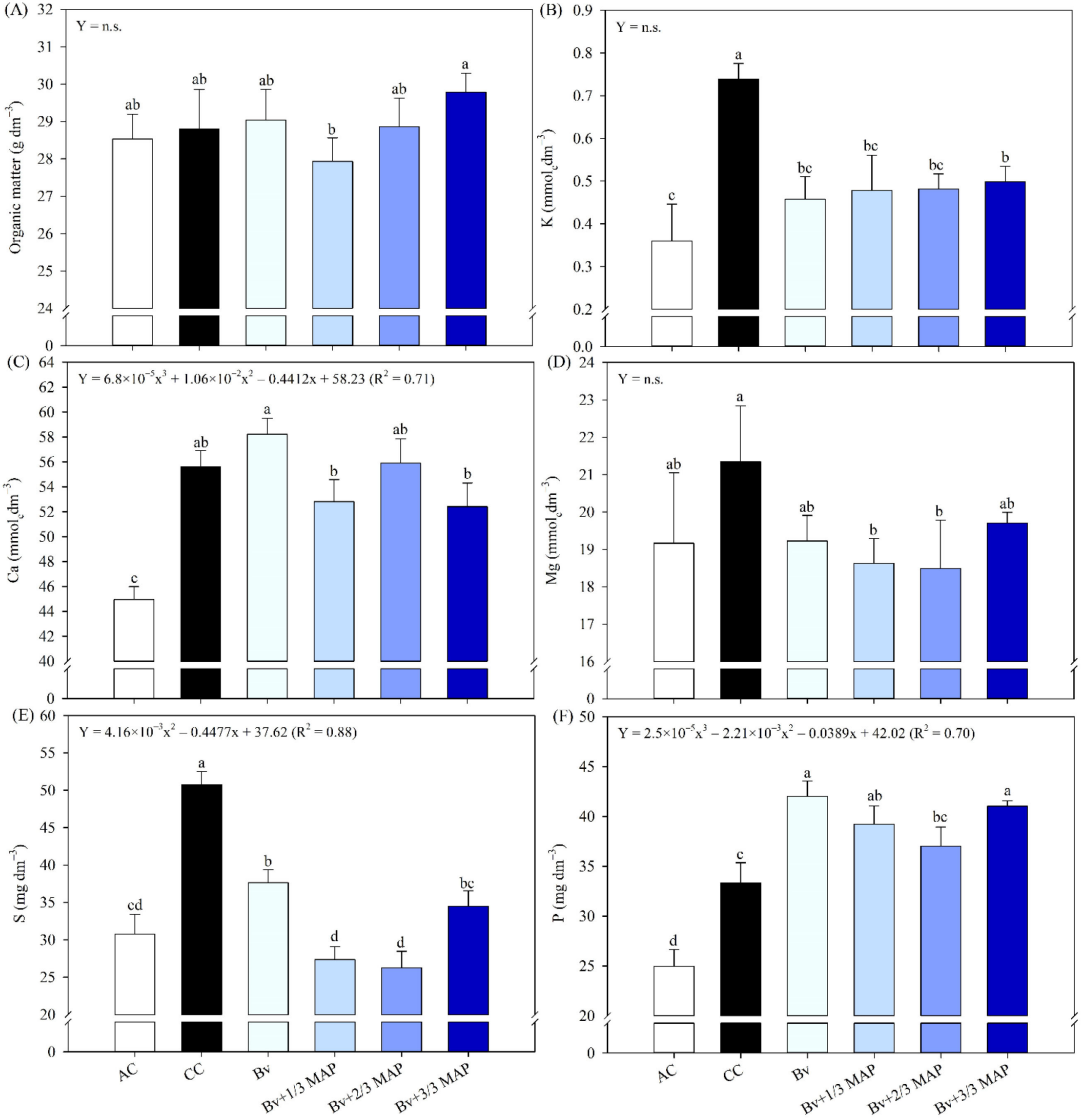
Organic matter **(A)**, potassium (K) **(B)**, calcium (Ca) **(C)**, magnesium (Mg) **(D)**, sulfur (S) **(E)**, and phosphorus (P) **(F)** contents in the soil after 180 days of sugarcane cultivation, under treatments with and without inoculation of *B. velezensis* UFV 3918 (Bv) and doses of mono ammonium phosphate (MAP). Means followed by the same letter do not differ at a 5% probability level, according to Tukey’s test. Error bars express the standard deviation of the mean (n = 4). Regression equations and R^2^ refer to the association between *B. velezensis* UFV 3918 and MAP doses at the 5% significance level (*). AC, absolute control; CC, commercial control.

**Figure 4:**
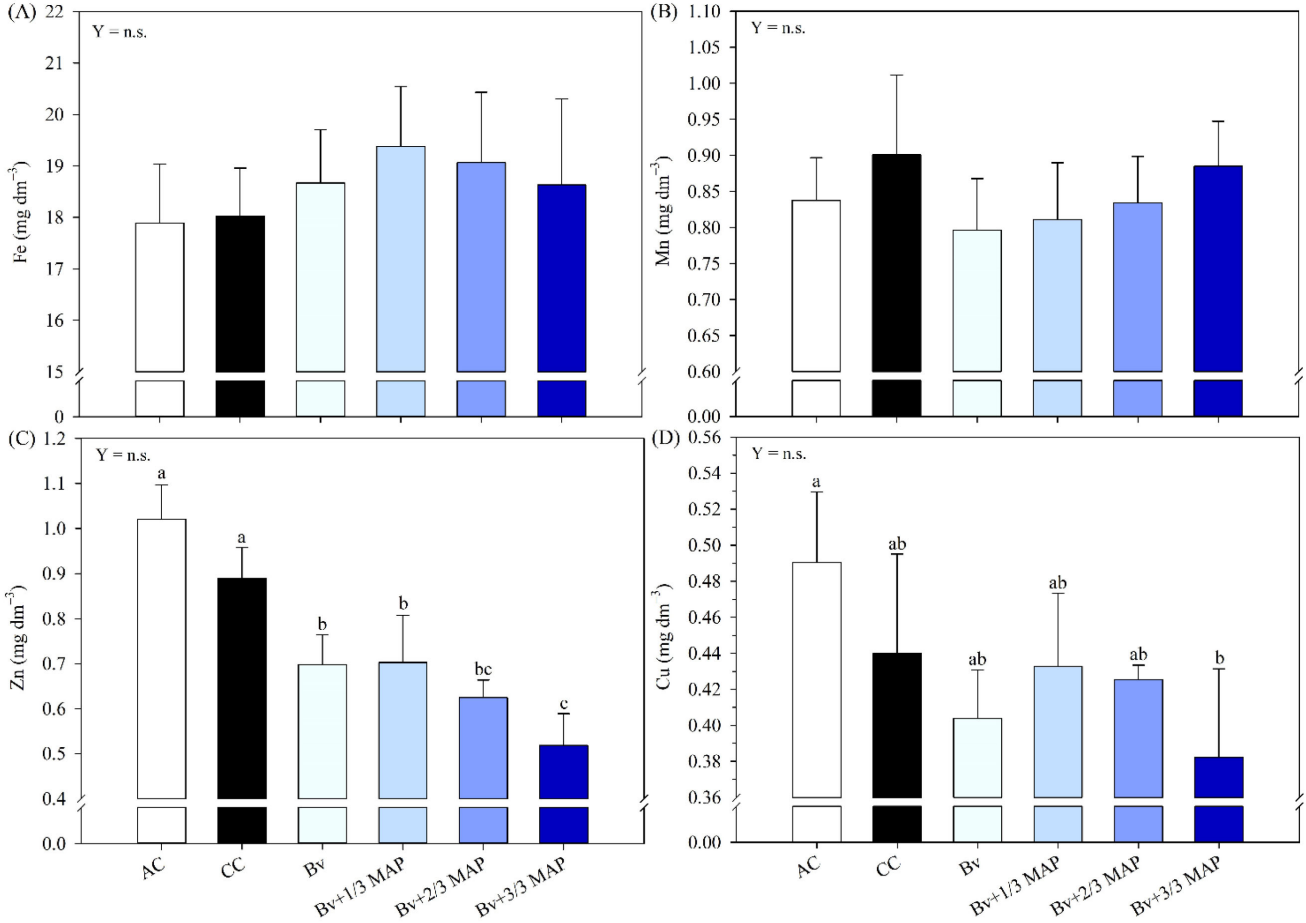
Iron (Fe) **(A)**, manganese (Mn) **(B)**, zinc (Zn) **(C)**, and copper (Cu) **(D)** contents in the soil after 180 days of sugarcane cultivation, under treatments with and without inoculation of *B. velezensis* UFV 3918 (Bv) and doses of mono ammonium phosphate (MAP). Means followed by the same letter do not differ at a 5% probability level, according to Tukey’s test. Error bars express the standard deviation of the mean (n = 4). Regression equations and R^2^ refer to the association between *B. velezensis* UFV 3918 and MAP doses at the 5% significance level (*). AC, absolute control; CC, commercial control.

No differences were observed between the *B. velezensis* treatment*s* and the CC for soil OM content (**Figure 3A**). In general, CC resulted in the highest soil contents of K (**Figure 3B**), S (**Figure 3E**), and Zn (**Figure 4C**), representing average increases of 54.2%, 61.5%, and 39.9%, respectively, compared with the inoculated treatments. No regression trends were observed for OM, K, or Zn as a function of Bv + MAP doses. However, soil S content decreased from 0 to 66% of the MAP dose, then increased slightly at the full MAP dose, indicating a quadratic response (0.88*) (**Figure 3E**).

Regardless of the MAP dose, there was no difference in Ca content between the inoculated treatments and CC (**Figure 3C**). Although it did not differ from Bv and Bv+3/3 MAP, CC provided an average increase of 14.5% in Mg content compared with Bv+1/3 and 2/3 MAP (**Figure 3D**). There was a tendency for Ca content to decrease with increasing MAP doses (0.71*). There was no adjustment in Mg content for Bv + MAP doses (**Figures 3C, D**).

Regarding P, the highest levels were observed in the inoculated treatments, with Bv and Bv+1/3 MAP providing an average increase of 22.0% in P content compared with CC (**Figure 3F**). Considering Bv + MAP doses, there was a reduction in P content from 0 to 66% of the MAP dose, followed by an increase in this nutrient content at 100% of the MAP dose (0.70*) (**Figure 3F**).

There was no difference between treatments for Fe (**Figure 4A**) and Mn (**Figure 4B**) contents. Regarding Cu, the highest content was found in AC, which differed only from Bv + 3/3 MAP, which had the lowest Cu content (**Figure 4D**). AC provided a 28.3% increase in Cu content compared with Bv + 3/3 MAP. There was no adjustment in Cu content for Bv + MAP doses.

### 3.4 Shoot nutrient accumulation

**Figure 5:**
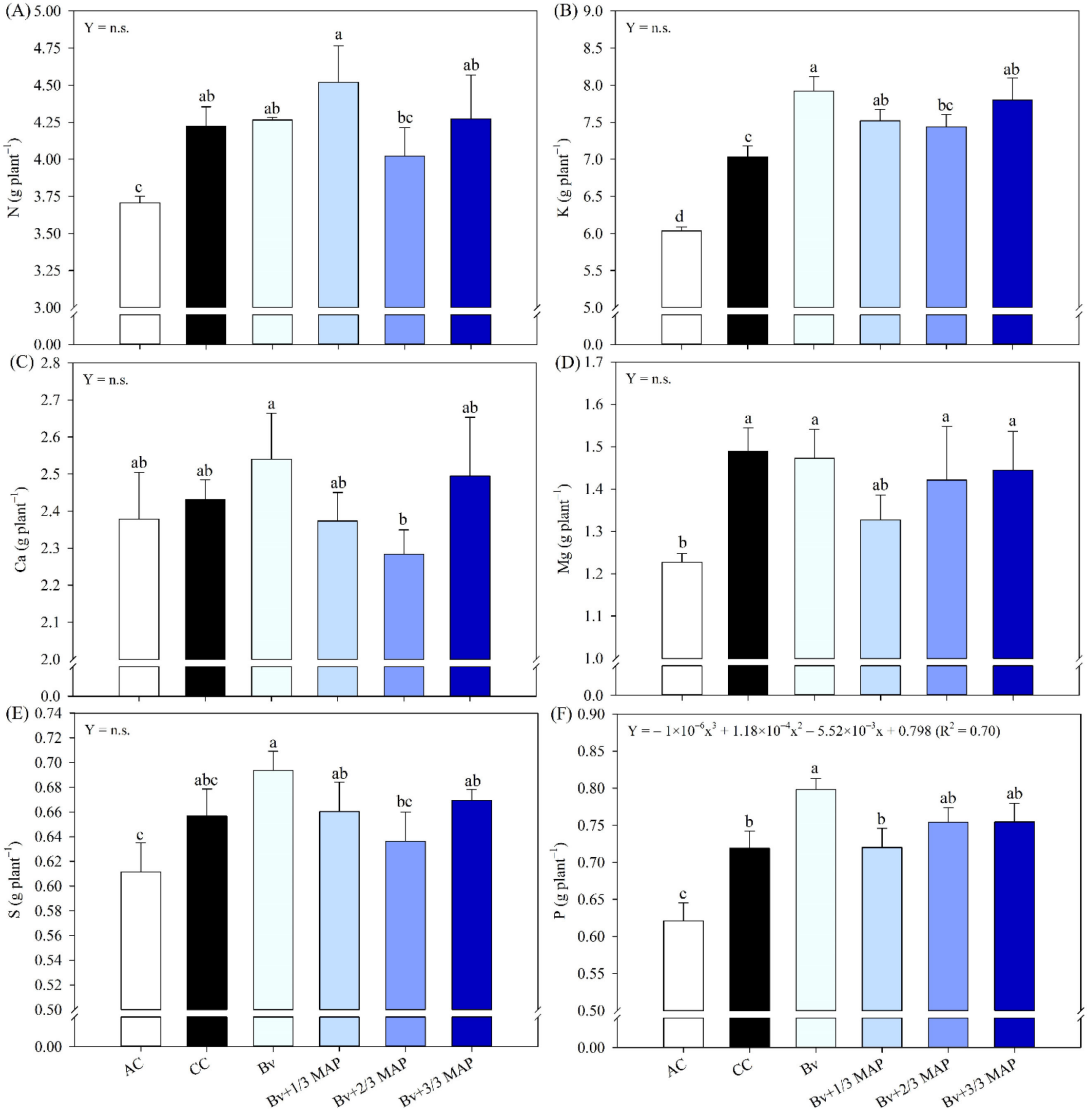
Sugarcane shoot accumulation of nitrogen (N) **(A)**, potassium (K) **(B)**, calcium (Ca) **(C)**, magnesium (Mg) **(D)**, sulfur (S) **(E)**, and phosphorus (P) **(F)**, under treatments with and without inoculation of *B. velezensis* UFV 3918 (Bv) and doses of mono ammonium phosphate (MAP). Means followed by the same letter do not differ at a 5% probability level, according to Tukey’s test. Error bars express the standard deviation of the mean (n = 4). Regression equations and R^2^ refer to the association between *B. velezensis* UFV 3918 and MAP doses at the 5% significance level (*). AC, absolute control; CC, commercial control.

**Figure 6:**
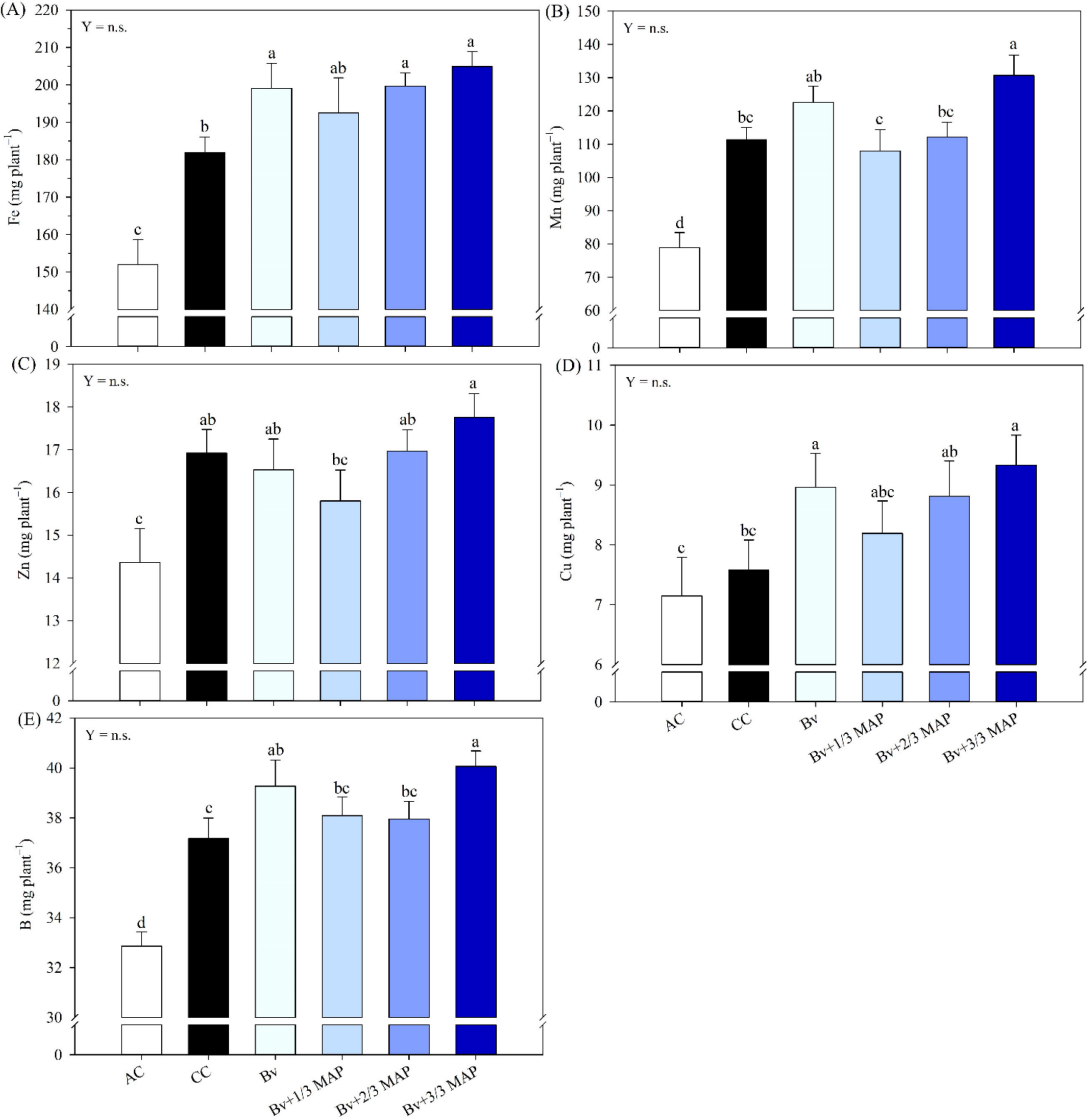
Sugarcane shoot accumulation of iron (Fe) **(A)**, manganese (Mn) **(B)**, zinc (Zn) **(C)**, copper (Cu) **(D)**, and boron (B) **(E)**, under treatments with and without inoculation of *B. velezensis* UFV 3918 (Bv) and doses of mono ammonium phosphate (MAP). Means followed by the same letter do not differ at a 5% probability level, according to Tukey’s test. Error bars express the standard deviation of the mean (n = 4). Regression equations and R^2^ refer to the association between *B. velezensis* UFV 3918 and MAP doses at the 5% significance level (*). AC, absolute control; CC, commercial control.

Regarding shoot nutrient accumulation (AcNu), the AC generally exhibited the lowest performance for both macro- and micronutrient (**Figures 5**, **6**). Bv and Bv+1/3 MAP promoted an average increase of 9.8% in AcK compared with the CC (**Figure 5B**). No significant differences were observed between the *B. velezensis*-inoculated plants and CC for AcN (**Figure 5A**), AcCa (**Figure 5C**), AcMg (**Figure 5D**), and AcS (**Figure 5E**). However, Bv alone resulted in a 5.6% increase in AcS compared with CC.

Although no differences were observed between the Bv+1/3, Bv+2/3, and Bv+3/3 MAP and the CC for AcP, Bv promoted increases of 11.0% and 28.5% in AcP compared with CC and AC, respectively (**Figure 5F**). When considering Bv + MAP doses, AcP showed a decreasing trend from 0 to 33% of the MAP dose, followed by a slight increase at 66% and 100% MAP, fitting a quadratic regression model (0.70*).

Bv, Bv+2/3 MAP, and Bv+3/3 MAP resulted in increases of 9.4%, 9.7%, and 12.6%, respectively, in AcFe compared with the CC (**Figure 6A**). For AcMn, Bv, Bv+1/3 MAP, and Bv+2/3 MAP showed similar performance to CC, whereas Bv+3/3 MAP led to a 17.3% increase in AcMn compared with CC (**Figure 6B**). No differences were observed between the inoculated plants and CC for AcZn (**Figure 6C**). Bv and Bv+3/3 MAP promoted average increases of 20.7% and 6.7% in AcCu and AcB, respectively, compared with CC (**Figures 6D, E**), while Bv+1/3 MAP and Bv+2/3 MAP did not differ from CC for either AcCu or AcB.

### 3.5 Biomass production

**Figure 7:**
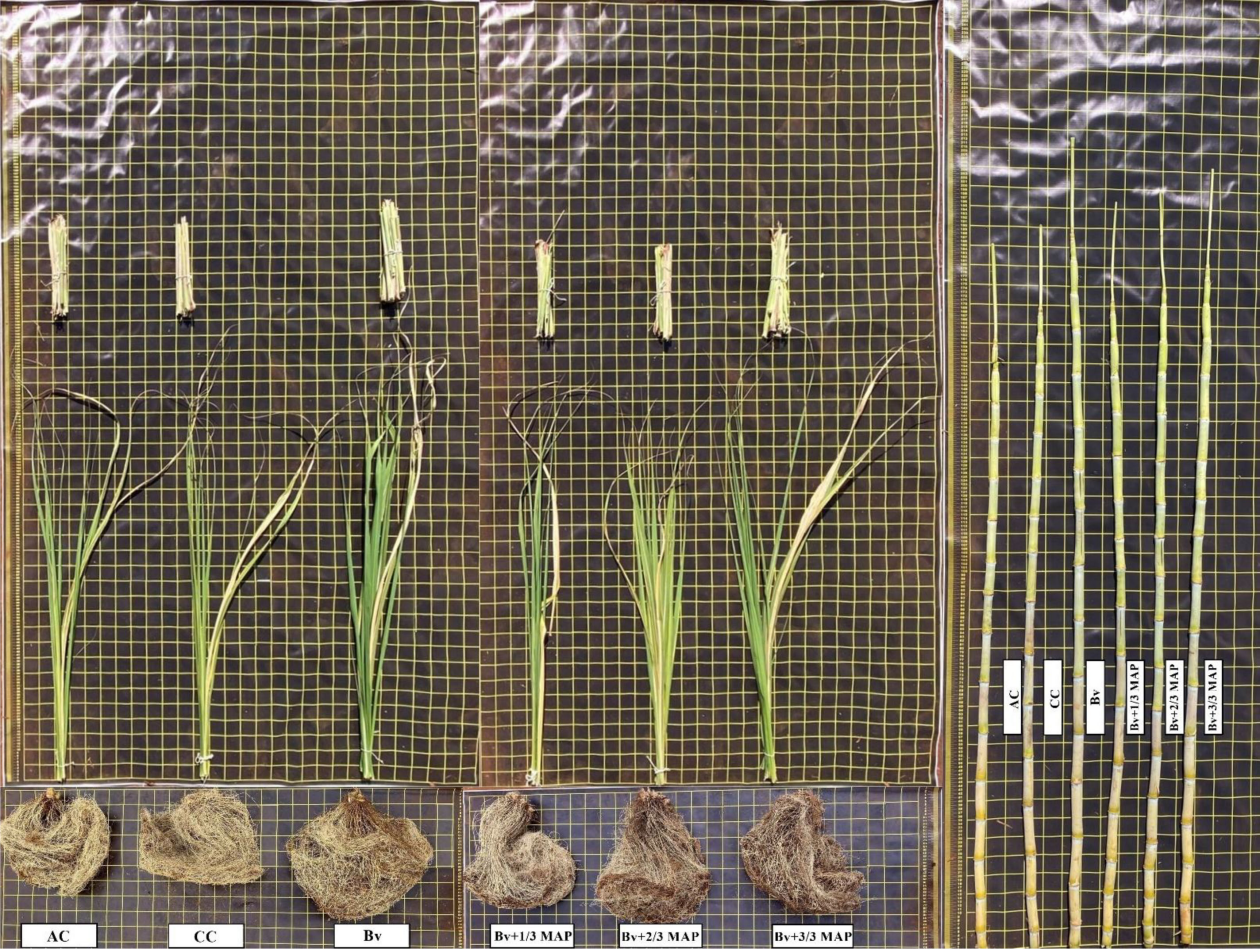
Shoot visual aspect of the main stalk (leaves, leaf sheaths, and stalks) and root system of sugarcane plants under treatments with and without inoculation of *B. velezensis* UFV 3918 (Bv) and doses of mono ammonium phosphate (MAP).

Sugarcane plants exhibited distinct biomass responses to *B. velezensis* inoculation and MAP application level, with a notable visual increase observed in plants inoculated with Bv and receiving no MAP (**Figure 7**).

**Figure 8:**
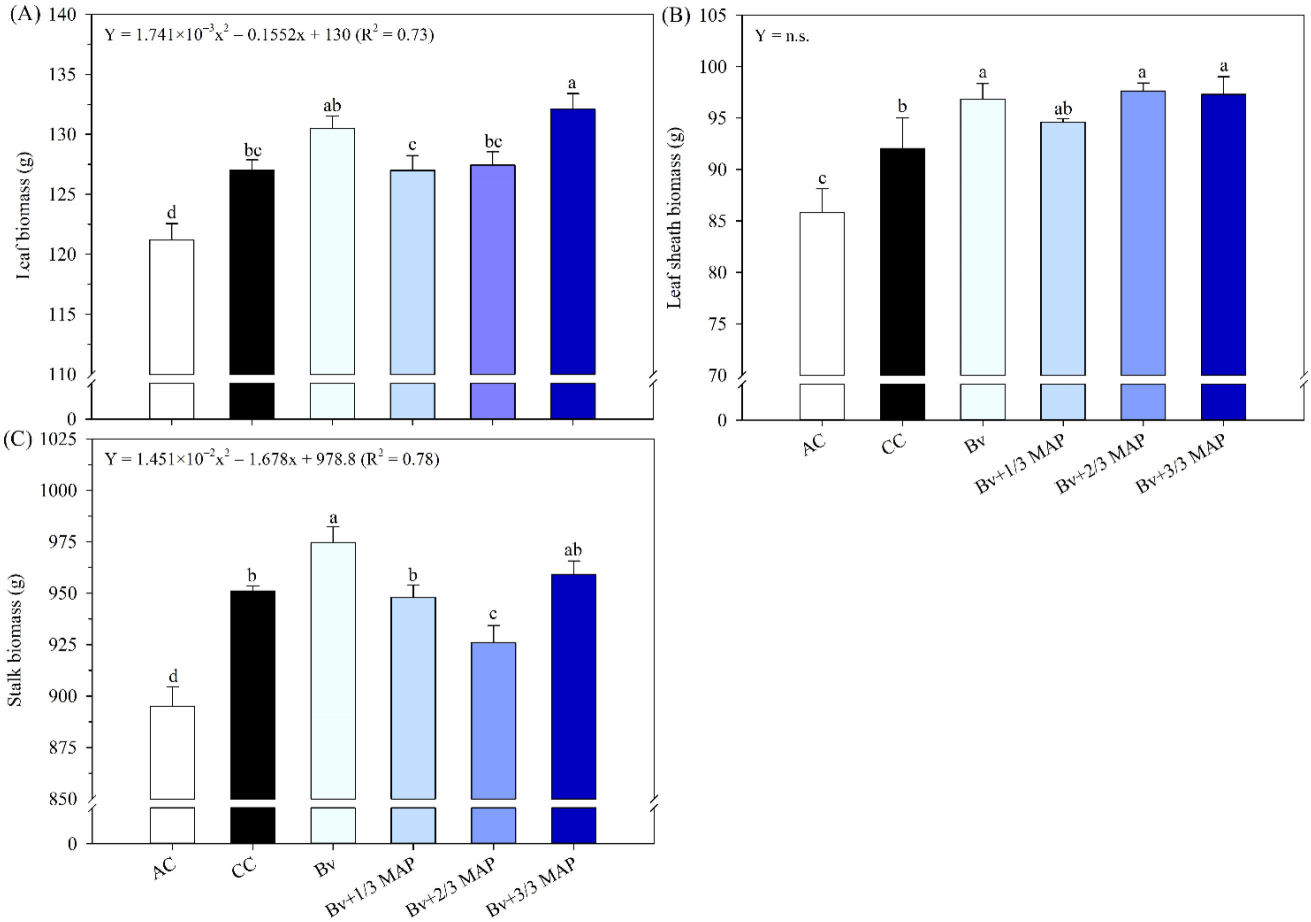
Biomass of leaves **(A)**, leaf sheaths **(B)**, and stalks **(C)** of sugarcane plants at 180 DAP, under treatments with and without inoculation of *B. velezensis* UFV 3918 (Bv) and doses of mono ammonium phosphate (MAP). Means followed by the same letter do not differ at a 5% probability level, according to Tukey’s test. Error bars express the standard deviation of the mean (n = 4). Regression equations and R^2^ refer to the association between *B. velezensis* UFV 3918 and MAP doses at the 5% significance level (*). AC, absolute control; CC, commercial control.

The highest LB was recorded in Bv+3/3 MAP, representing a 4.7% increase compared with the CC. However, LB under Bv+3/3 MAP was similar to that of Bv+1/3 MAP (**Figure 8A**). In general, increasing MAP doses in combination with Bv led to increased LB up to 100% of the MAP dose (0.73*), although the absence of MAP (Bv alone) yielded LB equivalent to the highest MAP dose, suggesting the potential for dose reduction.

There were no differences in LSB among the plants inoculated with *B. velezensis*. The highest LSB was observed in Bv, Bv+2/3 MAP, and Bv+3/3 MAP, which showed an average increase of 5.5% compared with CC (**Figure 8B**). There was no adjustment of Bv + MAP doses for LSB.

The highest SB values were observed under Bv and Bv+3/3 MAP. However, Bv+1/3 MAP resulted in SB similar to CC, reinforcing the hypothesis that inoculation with *B. velezensis* UFV 3918 can reduce P fertilization (**Figure 8C**). Bv alone led to a 3.8% increase in SB compared with CC. When considering the regression analysis, increasing MAP doses in combination with Bv resulted in a decline in SB up to 66% of the recommended MAP dose, followed by a slight increase at 100% (0.78*).

### 3.6 Principal component analysis and Pearson’s correlation

**Figure 9:**
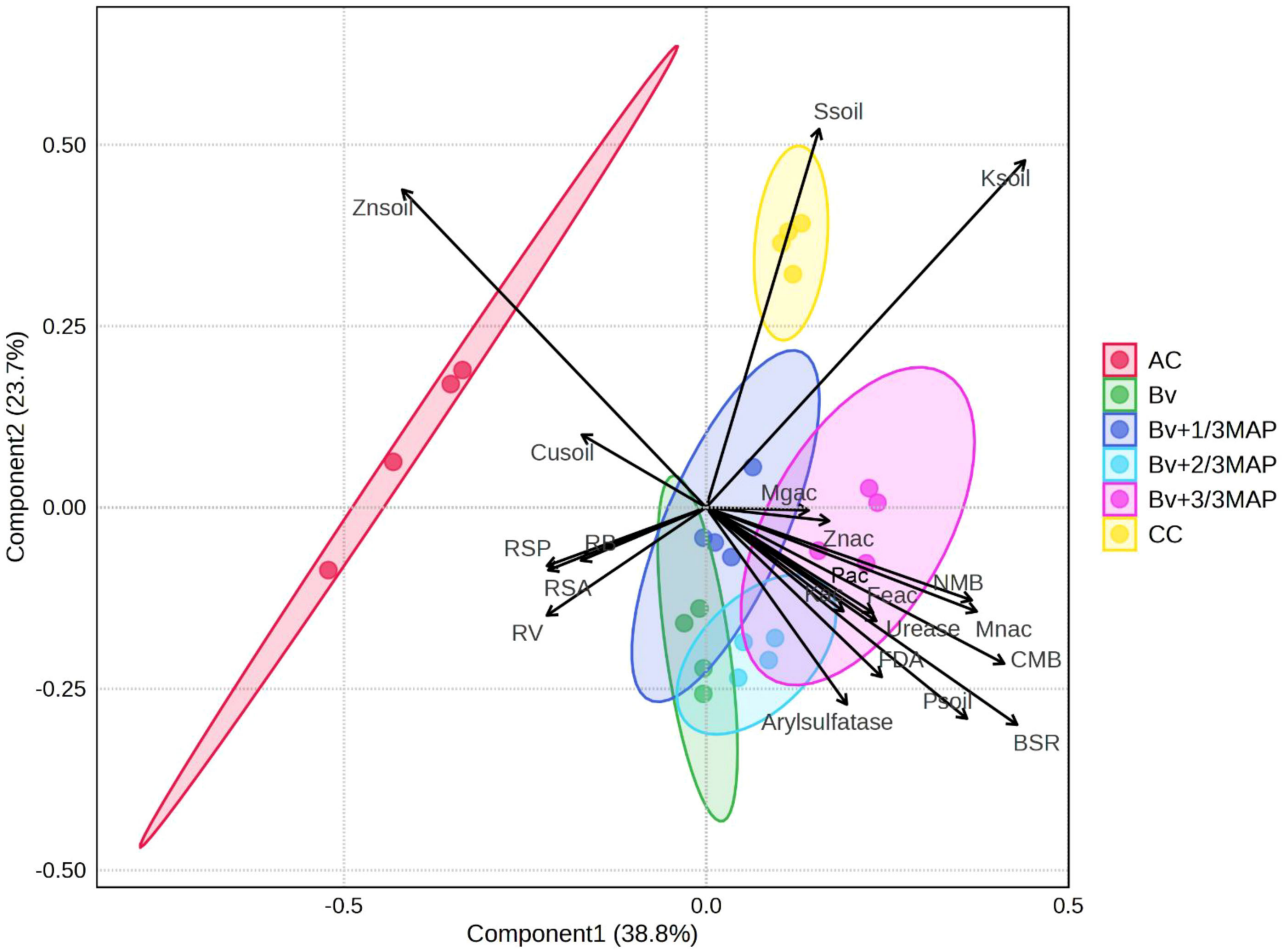
Biplot from Partial Least Squares Discriminant Analysis (PLS-DA), showing the distribution of treatments, the contribution of microbial, chemical, and plant variables to the discrimination among groups, and the relationships among soil microbial and chemical attributes, shoot nutrient accumulation, and sugarcane root traits under different treatments. Arrows indicate the direction and magnitude of each variable’s influence on the principal components. Treatments: AC, absolute control; CC, commercial control (3/3 MAP); Bv, *Bacillus velezensis* UFV 3918; Bv+1/3 MAP; Bv+2/3 MAP; Bv+3/3 MAP.

The first principal component (PC1) explained 38.8% of the data variability, while the second principal component (PC2) accounted for 23.7% (**Figure 9**; **Supplementary Figure 1**). AC was distinctly separated from the other treatments, indicating that its variable values differed substantially from those of *B. velezensis* and MAP treatments (**Figure 9**). This highlights the microorganism’s impact on the evaluated nutritional and microbiological attributes.

CC occupied an intermediate position, sharing some characteristics with *Bacillus* treatments but exhibiting a weaker effect (**Figure 9**). The *Bacillus* treatments (Bv, Bv+1/3 MAP, Bv+2/3 MAP, and Bv+3/3 MAP) clustered together, suggesting similar responses. Notably, Bv alone and in combination with 1/3 or 2/3 MAP showed proximity, suggesting that reducing MAP doses did not compromise the variables (**Figure 9**). This indicates the potential to reduce phosphate fertilizer use without adverse effects on soil and plant attributes.

AC was more strongly associated with Zn and Cu in the soil and root traits such as root biomass (RB), root volume (RV), root surface area (RSA), and root surface projection (RSP) (**Figure 9**). This suggests that in the absence of phosphate fertilization, root development and micronutrient accumulation were enhanced. CC was positioned separately and associated with soil K and S.

*Bacillus* treatments clustered near soil P, soil enzymes (arylsulfatase, FDA, and urease), basal soil respiration (BSR), microbial biomass nitrogen (NMB), microbial biomass carbon (CMB), and shoot nutrient accumulation (Mg, Zn, K, Fe, and Mn) (**Figure 9**). This indicates that *Bacillus*, particularly in combination with MAP, promoted P accumulation in the shoot and enhanced microbiological activity. Bv alone increased RSP, RB, RSA, and RV, which may have contributed to greater Zn, Mn, Fe, and P accumulation in the shoot, as well as increased soil P, though its impact was lower than that of *Bacillus* combined with MAP (**Figure 9**).

AC and CC showed distinct responses compared with *Bacillus* treatments. The application of *Bacillus* improved shoot P accumulation and microbiological activity, with Bv+2/3 MAP and Bv+3/3 MAP emerging as the most promising, as they correlated with key nutritional and microbiological variables (**Figure 9**).

**Figure 10:**
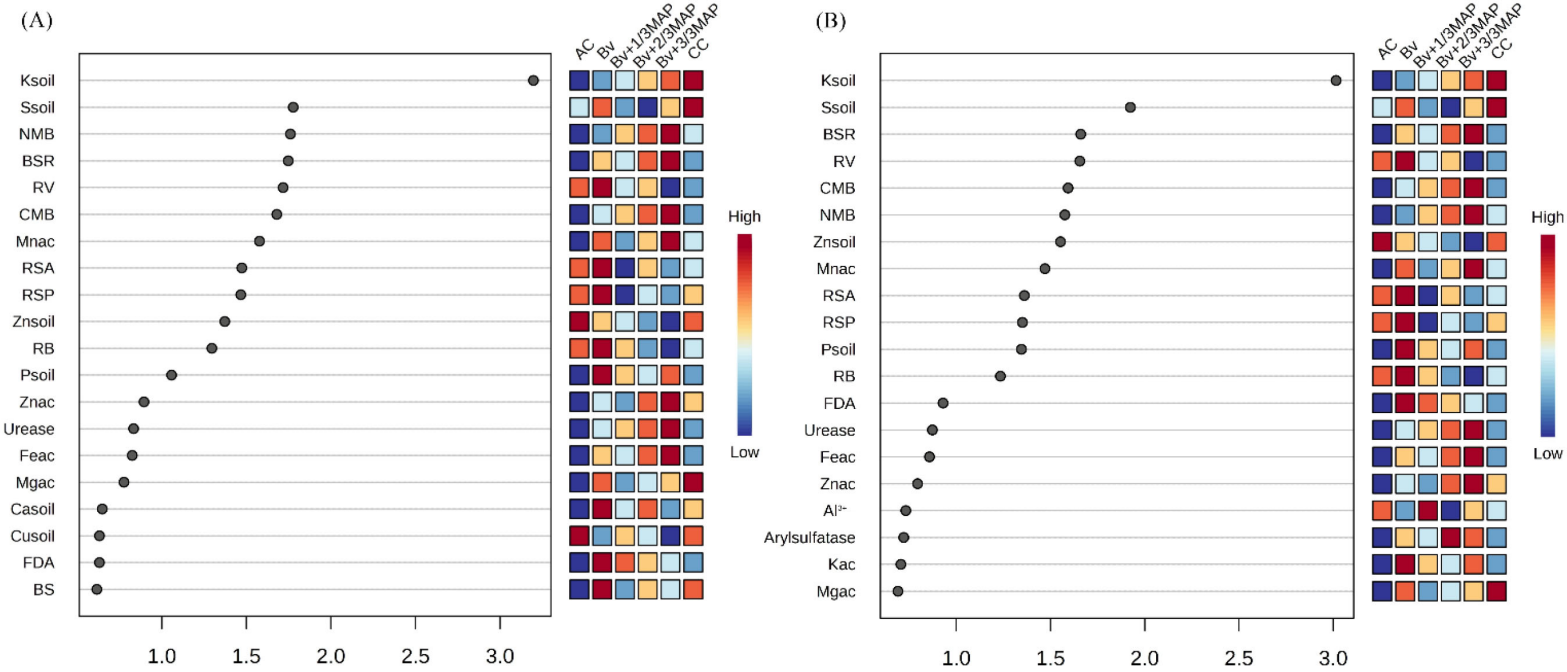
Variable Importance in Projection (VIP) scores derived from PLS-DA analysis for **(A)** PC1 and **(B)** PC2, indicating the contribution of soil and plant variables to the differentiation among treatments. Variables with VIP scores greater than 1.0 are considered the most influential in the model. Treatments: AC, absolute control; CC, commercial control (3/3 MAP); Bv, *Bacillus velezensis* UFV 3918; Bv+1/3 MAP; Bv+2/3 MAP; Bv+3/3 MAP.

In PC1 and PC2, the most influential variables in treatment differentiation were soil K and S, NMB, CMB, BSR, and RV (**Figure 10**). AC primarily enhanced root traits while negatively affecting nutritional and microbiological attributes. Bv+2/3 MAP and Bv+3/3 MAP increased soil K, NMB, CMB, and BSR, while Bv strongly promoted root traits, BSR, FDA, and soil P (**Figure 10**).

The differentiation of soil properties was strongly influenced by K and S, confirming the impact of phosphate fertilization on soil fertility (**Figure 9**. Additionally, microbial activity (NMB, BSR, CMB) was positively affected by Bv+MAP treatments, reinforcing their role in improving soil biological conditions.

The main variables influencing shoot P accumulation were soil P and Ca contents, soil enzyme activities (FDA, urease, β-glucosidase, arylsulfatase, and acid phosphatase), Fe and Mn accumulation, and the microbial indicators BSR, CMB, and NMB, with a marked contribution of *B. velezensis* in the absence of MAP fertilization (Bv) (**Supplementary Figure 2**).

**Figure 11:**
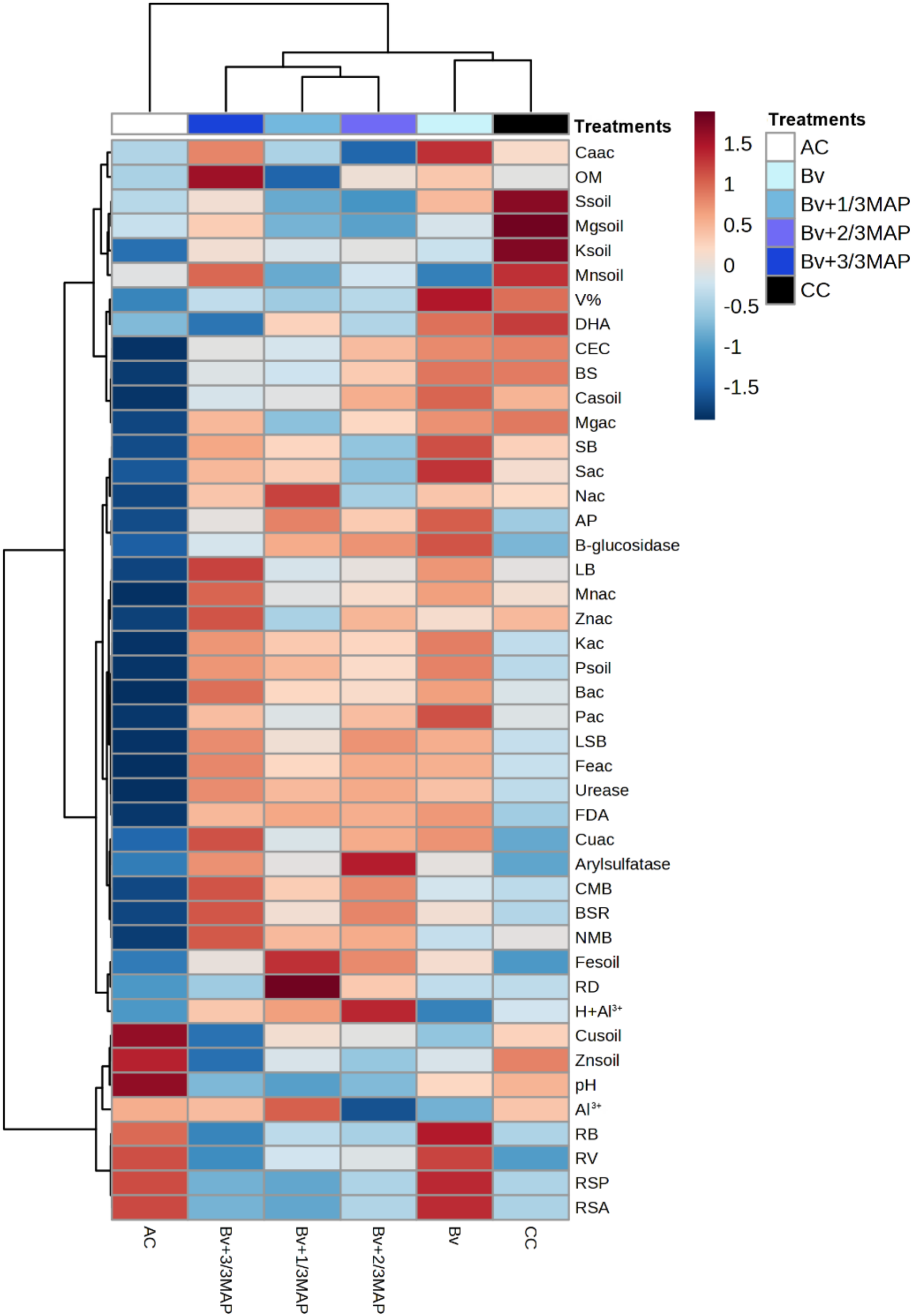
Heatmap of Pearson correlation coefficients between soil chemical and microbial properties, enzymatic activities, root traits, and shoot nutrient accumulation in sugarcane. Positive and negative correlations are indicated by a color scale from blue (negative) to red (positive).

AC showed the greatest reductions in nutritional and microbiological attributes while increasing soil Cu and Zn levels, as well as RB, RV, RSA, and RSP (**Figure 11**). Bv+1/3 MAP and Bv+2/3 MAP formed a distinct group, with Bv+3/3 MAP closely associated with them, while Bv exhibited similarities to CC.

The dendrogram indicates that microbiological indicators were key in soil P availability and shoot nutrient accumulation (**Figure 11**). Soil pH, Al^3+^, Zn, and Cu levels were closely linked to root traits. *Bacillus* treatments, particularly in combination with MAP, stimulated soil microbiota, increasing enzymatic activity and microbial biomass. This contributed to greater soil nutrient availability and enhanced shoot nutrient accumulation. The presence of Bv+MAP also contributed to pH neutralization, improving soil chemical conditions (**Figure 11**).

Bv enhanced the activity of dehydrogenase, acid phosphatase, β-glucosidase, urease, and FDA enzymes, leading to increases in V%, CEC, BS, SB, and soil Ca, Mg, and P contents (**Figure 11**). It also promoted shoot accumulation of Mg, S, Mn, K, P, B, Fe, and Cu. The lower initial P levels in Bv and AC treatments favored root development.

Enzymatic activities (FDA, urease, acid phosphatase – AP, arylsulfatase, and β-glucosidase) exhibited positive correlations with microbiological variables, including basal soil respiration (BSR), microbial biomass carbon (CMB), and microbial biomass nitrogen (NMB) (**Supplementary Figure 3**). This relationship suggests that increased enzymatic activity enhances soil microbial activity. Additionally, these microbiological variables were positively correlated with soil phosphorus content (Psoil), indicating a potential role of microorganisms in improving phosphorus availability (**Supplementary Figure 3**).

Root attributes, such as root volume (RV), root biomass (RB), and root surface projection (RSP), showed strong positive correlations among themselves and with soil Zn and Cu contents (**Supplementary Figure 3**). This finding suggests that root system development may be associated with the accumulation of these micronutrients in the soil. Conversely, these root traits exhibited negative correlations with microbiological indicators (CMB and BSR) and soil P content, suggesting that soils with greater microbial biomass and higher phosphorus availability may present lower root development (**Supplementary Figure 3**).

Soil pH was negatively correlated with soil phosphorus content (Psoil), indicating that phosphorus availability was higher in more acidic soils and lower in soils with elevated pH (**Supplementary Figure 3**). This effect may be attributed to the solubility of phosphorus-containing compounds, which tends to decrease under neutral to alkaline conditions due to precipitation with cations such as Ca and Mg.

Finally, the macro- and micronutrient contents in the sugarcane shoot, including Znac, Kac, and Feac, exhibited positive correlations with microbiological indicators and Psoil (**Supplementary Figure 3**). This suggests that enhanced microbial activity may improve nutrient uptake and utilization by the plant.

## 4 Discussion

The increasing global demand for food production requires a more intensive yet sustainable use of phosphate, a non-renewable and finite resource (Tonini et al., 2019). Fixed P accumulated through the indiscriminate use of phosphate fertilizers can be exploited by inoculating with phosphate-solubilizing bacteria (PSB) (Silva et al., 2023). In this research, we evaluated the inoculation of *B. velezensis* UFV 3918 as an alternative approach to reduce P fertilizer inputs in sugarcane cultivation. We investigated the effects of this strain on root development, soil chemical attributes, nutrient dynamics, and shoot plant performance.

### 4.1 Effect of microbiological indicators on P accumulation and soil health

Inoculation with *Bacillus velezensis* UFV 3918 stimulated soil microbial activity, as indicated by increases in soil basal respiration (BSR), fluorescein diacetate (FDA) hydrolysis, and microbial biomass (MBC and MBN). These responses suggest that the strain enhanced the metabolic activity and functional capacity of the soil microbiota, which likely contributed to improved nutrient cycling and P accumulation in sugarcane shoots.

Soil microbial communities play a central role in maintaining soil health by regulating key ecosystem processes, including nutrient cycling, biomass production, and microbiome stability (Yang et al., 2021; Wei et al., 2024). Inoculation with beneficial microorganisms, particularly *Bacillus* spp., can enhance root development and rhizodeposition, thereby shaping the composition and activity of the rhizosphere microbiota (Yahya et al., 2021). These interactions foster more efficient plant–microbe mutualisms (Latati et al., 2016; Bargaz et al., 2017) and contribute to improvements in microbial-driven processes such as BSR (Borden et al., 2021; Santos et al., 2022; Janati et al., 2023).

Among microbial indicators, BSR is widely recognized as a sensitive measure of microbial activity and overall soil biological quality, since it reflects changes induced by management practices (Brandán et al., 2016, 2017; Bünemann et al., 2018; Sáez-Sandino et al., 2023; Semenov et al., 2025). Similarly, FDA hydrolysis is a reliable indicator of total microbial activity, encompassing the action of various enzymes such as esterases, lipases, and proteases (Adam and Duncan, 2001; Ding et al., 2020). In our study, the strong positive correlation between microbiological indicators and enzymatic activity suggests that the observed increases in BSR and FDA under inoculation were associated with enhanced acid phosphatase activity and, consequently, improved P solubilization and shoot accumulation, particularly in the absence of MAP. This likely explains why BSR and FDA were among the most critical factors differentiating treatments.

These results are consistent with Sabaté and Brandán (2022), who reported similar increases following inoculation with *B. amyloliquefaciens* B14, and with Santos et al. (2022), who showed that *B. velezensis* UFV 3918 combined with organomineral fertilizer and MAP enhanced both BSR and FDA activity, particularly at higher MAP doses. The elevated microbial and enzymatic activity observed here likely contributed to greater mineralization of organic matter and the release of essential nutrients, fostering microbial proliferation and improving soil fertility (Bahram et al., 2018; Li et al., 2024a), processes that are tightly regulated by microbial abundance and community structure (Siwik-Ziomek and Szczepanek, 2019; Li et al., 2022). Moreover, the well-documented benefits of *Bacillus* inoculation, including increased microbial diversity and higher relative abundance of beneficial taxa in the rhizosphere (Han et al., 2019; Azeem et al., 2021; Bhattacharya et al., 2024; Jiang et al., 2024), support the idea that the observed changes in soil microbiological indicators in our trial are a direct result of inoculation, highlighting the practical potential of *B. velezensis* UFV 3918 to improve soil health and nutrient availability under sugarcane cultivation.

Microbial biomass carbon (MBC) serves as an integrative proxy for overall microbial activity and nutrient cycling in soil (Farrell et al., 2014; Fraser et al., 2016; Shi et al., 2024). Here, inoculation with *B. velezensis* UFV 3918 increased MBC, with the highest values observed at higher MAP doses, highlighting the interactive effect of microbial inoculation and P fertilization. Similarly, microbial biomass nitrogen (MBN) increased with *B. velezensis* inoculation at higher MAP levels, indicating a more active microbial community capable of supporting nutrient cycling and plant nutrition. These findings are consistent with previous studies reporting enhanced microbial biomass following *Bacillus* inoculation (Sabaté et al., 2020; Azeem et al., 2021; Sabaté and Brandán, 2022; Santos et al., 2022; Peng et al., 2025), but importantly, our results highlight the direct effect of the inoculant under different fertilization regimes.

The increases in MBC and MBN observed in this study are likely linked to enhanced availability of organic substrates, especially C and N inputs (Mason-Jones et al., 2023), including root exudates, which stimulate microbial proliferation and enzymatic activity. This suggests that inoculation with *B. velezensis*, particularly in combination with MAP, promotes both microbial growth and nutrient turnover, reinforcing its potential to improve soil health and P availability (Wang et al., 2019a).

Soil enzymes act as early indicators of microbial functionality and environmental changes, mediating the transformation and mineralization of organic matter and nutrients (Kong et al., 2014; Xie et al., 2017; Semenov et al., 2025). In our study, urease, a key enzyme in the nitrogen cycle, exhibited increased activity in the inoculated treatments compared with the uninoculated treatment, indicating enhanced nutrient availability for plants and potential growth promotion (Wu et al., 2019). However, unlike MBN, urease activity did not respond directly to increasing MAP doses, suggesting that its stimulation is more associated with the structure of the microbial community than with fertilization per se. Similar increases in urease activity following *Bacillus* inoculation have been reported by Duan et al. (2022) and Santos et al. (2022).

β-Glucosidase, an enzyme involved in cellulose degradation and C cycling, was enhanced only by *B. velezensis* inoculation in the absence of MAP, with no synergistic effect observed at higher P doses. Interestingly, while β-glucosidase activity is generally correlated with MBC (Almeida et al., 2015), in our study, higher MAP doses increased MBC but reduced this enzyme’s activity. This apparent decoupling suggests that elevated P availability may shift microbial metabolic pathways toward alternative enzymatic routes or reduce the need for organic matter decomposition, thereby lowering β-glucosidase expression.

Arylsulfatase plays a central role in the sulfur cycle by catalyzing the hydrolysis of organic sulfate esters and can be a limiting step for S mineralization in soils (Li and Sarah, 2003; Jung et al., 2012). Its activity is closely linked to sulfur and phosphorus dynamics in the rhizosphere. Phosphate has a higher affinity for soil mineral surfaces than sulfate, often displacing sulfate ions from exchange sites and reducing S availability (Kaiser et al., 1996; Alvarez et al., 2007; Assefa et al., 2021). This competition explains the inverse spatial pattern observed between soil S and P contents (**Figure 10**). In our study, increasing MAP doses reduced soil S availability, likely triggering a physiological response in the soil microbiota, increasing arylsulfatase activity as a compensatory mechanism to access organic S sources (Wang et al., 2019b). Thus, elevated arylsulfatase activity under higher MAP doses may reflect microbial adaptation to intensified sulfur limitation.

Acid phosphatase (AP) and dehydrogenase (DHA) activities, commonly used to measure soil biological activity and nutrient cycling, were also strongly influenced by *B. velezensis* inoculation. Several studies have reported enhanced activity of these enzymes following bacterial inoculation (Sabaté et al., 2020; Sabaté and Brandán, 2022; Santos et al., 2022). Acid phosphatases catalyze the hydrolysis of organic phosphorus compounds into forms available for plant uptake (Krämer and Green, 2000; Chen and Arai, 2023). In our experiment, *B. velezensis* inoculation resulted in higher AP activity than uninoculated treatments, highlighting its capacity to stimulate P mineralization. However, AP activity declined with increasing MAP doses, consistent with the well-documented negative feedback regulation of phosphatase enzymes by inorganic P availability. While N addition can stimulate phosphatase synthesis (Heuck et al., 2018), elevated soil P levels inhibit enzyme production by downregulating microbial gene expression associated with P acquisition (Janes-Bassett et al., 2022).

This feedback mechanism is supported by our data, which showed reductions in both absolute and specific AP activity with increasing P availability (**Figure 1**2), aligning with findings from Fraser et al. (2015); Zhang et al. (2015), and Xie et al. (2021). Similar responses were observed by Lopes et al. (2021) and Santos et al. (2022) in sugarcane systems, in which AP activity declined with increasing P input. A comparable trend was observed for DHA activity, an intracellular enzyme that reflects the metabolic activity of viable microbial cells (Skujins, 1973). Despite being less directly involved in P cycling, DHA activity also decreased with higher MAP doses, suggesting that high P availability may reduce overall microbial activity or shift microbial metabolism away from nutrient acquisition. Our previous studies involving *B. velezensis* UFV 3918 inoculation combined with organomineral fertilizer and MAP also showed reduced DHA activity under high P supply (Santos et al., 2022). Taken together, these results reinforce the idea that excessive fertilization can suppress enzymatic indicators of soil health, even when beneficial microbial inoculants are applied.

### 4.2 *B. velezensis* modulates root architecture in P-rich soil

Rhizosphere microorganisms associated with plant roots can influence root system architecture through multiple mechanisms, enhancing nutrient uptake, particularly under nutrient-limiting conditions (Hussain et al., 2019; Emami et al., 2019; Li et al., 2021, 2024). Inoculation with phosphate-solubilizing bacteria (PSB) can stimulate the production of P-hydrolyzing enzymes and phytohormones, thereby promoting root growth and modifying root architecture to improve phosphorus acquisition (Batool and Iqbal, 2019; Suleman et al., 2018; Emami et al., 2019; Hashem et al., 2019). Among these phytohormones, indole-3-acetic acid (IAA) increases the number of basal and lateral roots (Lim and Kim, 2009; Zhang et al., 2022), while gibberellic acid (GA) promotes lateral root elongation (Joo et al., 2004). *Bacillus velezensis* UFV 3918 produces both IAA and GA, which likely underlie the observed increases in root length (RL), root volume (RV), and root biomass (RB). These traits were particularly important in differentiating the treatments, as phytohormones are the main microbial signals regulating root system architecture (Li et al., 2024b).

Phosphate fertilization can select for specific bacterial communities (Grafe et al., 2018; Tian et al., 2022) and regulate genes involved in microbial P cycling, often reducing PSB competitiveness in P-rich soils (Long et al., 2018; Widdig et al., 2019; Dai et al., 2020). Several studies report that PSB activity is higher in unfertilized soils than in high-P environments. Consistent with this, in our study, the highest RL, RV, and RB were observed in plants inoculated with *B. velezensis* alone (Bv) or with the lowest MAP dose (Bv+1/3 MAP). These values declined with increasing MAP doses, supporting the hypothesis that higher P availability negatively affects *Bacillus* activity and its growth-promoting effects. Root systems with reduced primary root elongation and enhanced lateral root formation near the soil surface confer advantages in accessing immobile nutrients such as phosphorus (Wang et al., 2015; Lynch, 2019). RV was a critical variable for P accumulation, with inoculated plants showing up to 30.5% higher RV than the uninoculated control (3/3 MAP).

These results are consistent with previous studies reporting that auxin-producing PSB strains alter sugarcane root architecture and enhance RL, RV, RB, root surface area, and nutrient uptake (Santos et al., 2019; Safirzadeh et al., 2019). Araujo et al. (2021) similarly reported increases in RL and RV in soybean plants inoculated with *B. subtilis*. The negative correlations observed between root traits, microbiological indicators, and soil P content suggest that biologically active soils may reduce the need for an extensive root system, possibly due to enhanced nutrient availability and delivery efficiency. Interestingly, the uninoculated low-P treatment (AC) exhibited root development patterns similar to those of Bv, likely due to P limitation, whereas the nutritional benefits observed in inoculated plants were absent in AC.

### 4.3 Soil P, Ca, and S availability influence shoot P accumulation

The biplot analysis revealed that increased soil P levels were associated with decreased soil S content, and that higher soil P and Ca concentrations, combined with reduced S availability, were key drivers of enhanced shoot P accumulation in inoculated plants. A positive correlation was observed between soil P and Ca contents and shoot P accumulation (**Figure 9**, **Supplementary Figure 3**). Phosphorus and Ca are essential for robust root proliferation (Rane et al., 2015), with P playing a crucial role in root development, anatomical modifications, and root hair density, all contributing to increased sugarcane yield (Kingston, 2014; Elhaissoufi et al., 2020). Conversely, Ca deficiency is commonly associated with restricted root growth (Kingston, 2014). Inoculation with *B. velezensis* enhanced soil P availability, which, in combination with Ca, stimulated root development in inoculated plants (Bv), improving P uptake and resulting in greater shoot P accumulation. However, the application of concentrated P fertilizers, such as monoammonium phosphate (MAP), can induce S deficiency due to nutrient antagonism, as the interaction between P and S affects their respective critical thresholds in the soil (Alvarez et al., 2007; Malhotra et al., 2018). This likely explains the reduced soil S content observed in inoculated treatments compared with the control without MAP (AC), reflecting the enhanced microbial activity and nutrient demand associated with plant–microbe interactions.

### 4.4 Enhanced nutrient availability and biomass production

The release of organic acids into the rhizosphere is a primary mechanism for phosphate solubilization (Rfaki et al., 2020; Leite et al., 2024). However, in this study, soil solution pH remained stable after inoculation (mean = 6.4), indicating that phosphate solubilization can occur without a reduction in pH (Marra et al., 2011). The observed increase in P availability may instead be attributed to the mineralization of organic phosphorus, supported by the elevated activity of acid phosphatase in inoculated treatments, which hydrolyzes phosphoester and phosphoanhydride bonds to release bioavailable P. Improvements in RV, RSA, RSP, and RB in AC and Bv treatments were linked to enhanced root architecture, reflecting both adaptive responses to low P availability (Soumya et al., 2021) and bacterial stimulation of root growth (Hussain et al., 2019; Emami et al., 2019), ultimately favoring P acquisition.

Although higher soil pH typically reduces P availability through precipitation with calcium and magnesium, forming insoluble complexes (Penn and Camberato, 2019), the microbial community can mitigate these effects. *Bacillus* spp. can release organic acids that solubilize phosphorus even under suboptimal pH conditions (Khan et al., 2024). Inoculation has also been reported to enhance the uptake and accumulation of macronutrients (N, P, K, Ca, Mg) in rice (Costa et al., 2015), sugarcane (Cipriano et al., 2021), and common bean (Hassan et al., 2017; Bamagoos et al., 2021), as well as micronutrients such as Fe, Mn, Zn, and Cu in sugarcane (Labanca et al., 2020), common bean (Bamagoos et al., 2021), maize (Goteti et al., 2013), tomato (Liu et al., 2020), and wheat (Rana et al., 2012).

Magnesium is central for activating kinases and facilitating phosphate group transfer (Malhotra et al., 2018), but nutrient interactions can lead to antagonistic effects. For example, high P levels may suppress Zn uptake by downregulating Zn transporters and promoting Zn immobilization in roots via phytate formation (Huang et al., 2000; Ova et al., 2015; Zhang et al., 2016, 2017). This is consistent with the lower soil Zn content observed in inoculated treatments.

Inoculated plants also exhibited higher shoot concentrations of K, Fe, B, and Cu compared with non-inoculated controls, all of which are essential for photosynthesis and biomass accumulation. Potassium regulates stomatal opening, turgor, CO_2_ assimilation, and phloem sucrose translocation (Rodrigues et al., 2018), while boron contributes to sugar transport, carbohydrate metabolism, cell wall structure, lignification, and auxin regulation (Kirkby and Römheld, 2004; Rodrigues et al., 2018). Iron is critical for chloroplast development, chlorophyll biosynthesis, electron transport, and redox functions (Marschner, 1995; Ma et al., 2019), and copper, a component of plastocyanin and other proteins, supports the photochemical phase of photosynthesis and CO_2_ fixation (Sykes, 1985; Ma et al., 2019).

Beyond phosphate solubilization, PSBs can promote plant growth by producing phytohormones and secondary metabolites (Tahir et al., 2017; Silva et al., 2023). *Bacillus* synthesizes auxins, gibberellins, and expansins, which enhance cell and plant growth (Zubair et al., 2019), likely contributing to the increased stalk biomass observed in *B. velezensis*-inoculated plants, particularly when combined with reduced MAP application (Bv and Bv+1/3 MAP). From a practical perspective, using *B. velezensis* with reduced MAP doses effectively maintained soil fertility and plant nutritional status without compromising biomass production or overall crop performance.

### 4.5 Importance of micronutrients in shoot P accumulation

Red Latosols are typically rich in Fe (Santos et al., 2018), and their highly weathered nature leads to the fixation of much of the applied phosphate fertilizer by Fe, Al, and Mn oxides and hydroxides, which are not easily released through simple desorption (Sato and Comerford, 2006; Bindraban et al., 2020). Consequently, Fe and Mn levels strongly influence P availability, explaining the negative correlation observed between soil Mn content and shoot P accumulation. In our study, inoculation with *B. velezensis* increased both soil P availability and shoot P accumulation compared with non-inoculated treatments, highlighting the strain’s capacity to enhance nutrient uptake under these conditions.

Although antagonistic interactions between P and Fe/Mn availability have been reported (Barben et al., 2010; Meng et al., 2021), such effects were not evident in our results. Instead, inoculated plants showed increased accumulation of both Fe and Mn in the shoots, which likely contributed to the observed enhancement in P uptake. This suggests that UFV 3918 possesses mechanisms that improve the availability and acquisition of these micronutrients. *Bacillus* species produce siderophores, low-molecular-weight, high-affinity Fe-chelating compounds, which increase Fe solubility in the rhizosphere, restrict competition from other microorganisms for Fe, and promote plant growth (Andrews et al., 2003; Schiessl et al., 2017; Pandey et al., 2017). Siderophores may also facilitate Mn mobilization and contribute to P solubilization (Gontia-Mishra et al., 2016; Sharma et al., 2013).

In addition to microbial mechanisms, sugarcane roots release phytosiderophores (Strategy II), chelating Fe^3+^ for uptake via specific plasma membrane transporters, functionally analogous to microbial siderophore-mediated acquisition (Römheld and Marschner, 1986; Römheld, 1991; von Wirén et al., 1993). Some rhizobacteria, including *Bacillus* spp., can also reduce Mn^4+^ to bioavailable Mn^2+^ in the rhizosphere through redox reactions facilitated by root exudates and organic matter decomposition (Dotaniya and Meena, 2015; Marschner, 1995).

Consistent with these mechanisms, our study confirmed that *B. velezensis* UFV 3918 produces siderophores (**Supplementary Figure 4**), as previously reported for other strains (Chen et al., 2019; Wei et al., 2020; Hasan et al., 2022; Mahdi et al., 2022; Mosela et al., 2022). The increased accumulation of Fe and Mn in inoculated plants is therefore likely attributable to siderophore production, supporting both micronutrient uptake and enhanced shoot P accumulation. These results emphasize the direct contribution of UFV 3918 to nutrient acquisition, rather than merely reflecting general microbial effects reported in the literature.

In conclusion, this study demonstrated that *Bacillus velezensis* UFV 3918 effectively enhanced nutrient uptake and accumulation in sugarcane, particularly phosphorus (P), while improving soil microbiological and chemical quality. Inoculation, whether applied alone or combined with reduced monoammonium phosphate (MAP) doses (Bv, Bv+1/3 MAP, Bv+2/3 MAP), increased soil basal respiration and the activity of key enzymes, including FDA, urease, β-glucosidase, and acid phosphatase. These microbial enhancements promoted root development and increased soil P availability, with acid phosphatase activity playing a central role in facilitating P solubilization and shoot accumulation. The high initial P content allowed *B. velezensis* to maintain adequate P supply even in the absence of phosphate fertilization, demonstrating its efficiency in mobilizing P for plant uptake.

Inoculation also increased shoot concentrations of K, Mn, Zn, Fe, Cu, and B, highlighting the strain’s role in improving micronutrient acquisition, particularly Fe and Mn, which are closely associated with organic matter and microbial activity. The observed increase in shoot Fe supports the strain’s siderophore production capacity, reinforcing its multifunctional role in promoting plant growth.

These findings have practical and sustainable implications, as P is often limited in tropical soils and critical for long-term sugarcane productivity. Therefore, *B. velezensis* UFV 3918 represents a promising tool to optimize phosphate fertilizer use in sugarcane cultivation, reducing reliance on mineral inputs while maintaining soil fertility, plant nutrition, and crop yield.

## Data Availability

The data presented in this study are deposited in the UNESP repository, accession number S237s, available at http://hdl.handle.net/11449/242558. The data are also available from the corresponding author upon reasonable request.
