# Supplementary material for: Enhancing soil health with Bacillus velezensis UFV 3918 boosts phosphorus and nutrient accumulation in sugarcane
Source: Front Plant Sci. 2026 Apr 23;17:1805752. doi: 10.3389/fpls.2026.1805752 (PMC13149259; doi:10.3389/fpls.2026.1805752)
Supplement: Supplementary file 1 [file DataSheet1.docx]

Supplementary Material

# Supplementary Figures


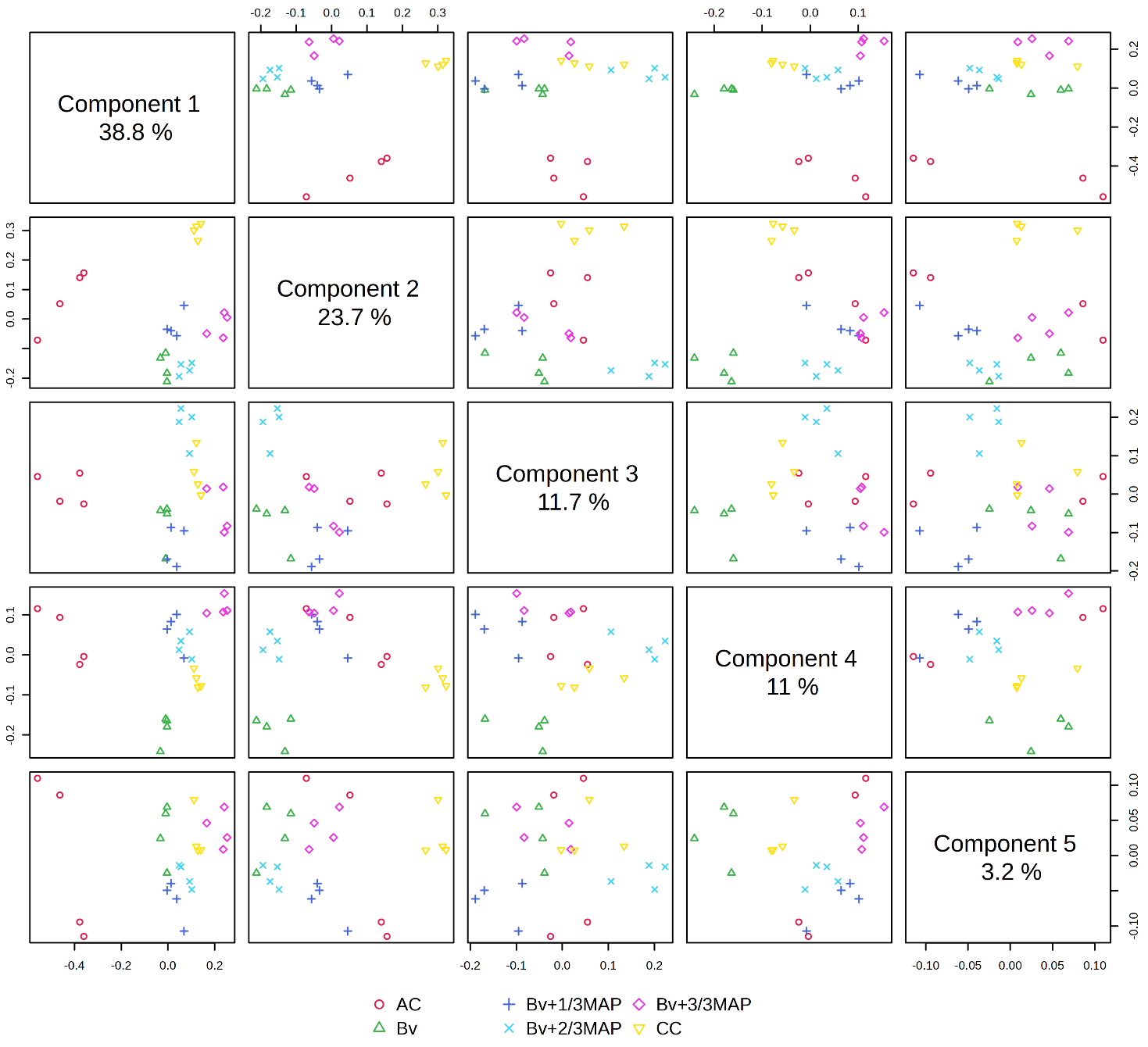
**Supplementary Figure 1.** Principal component analysis (PCA) of soil chemical and microbiological properties and shoot nutrient accumulation in sugarcane, as influenced by Bacillus velezensis UFV 3918 inoculation and different doses of monoammonium phosphate (MAP). The first principal components (PC1 and PC2) explain 38.8% and 23.7% of the total variance, respectively. Treatments: AC – absolute control (no MAP), CC – commercial control (recommended MAP dose), Bv – B. velezensis, Bv+1/3 MAP, Bv+2/3 MAP, and Bv+3/3 MAP


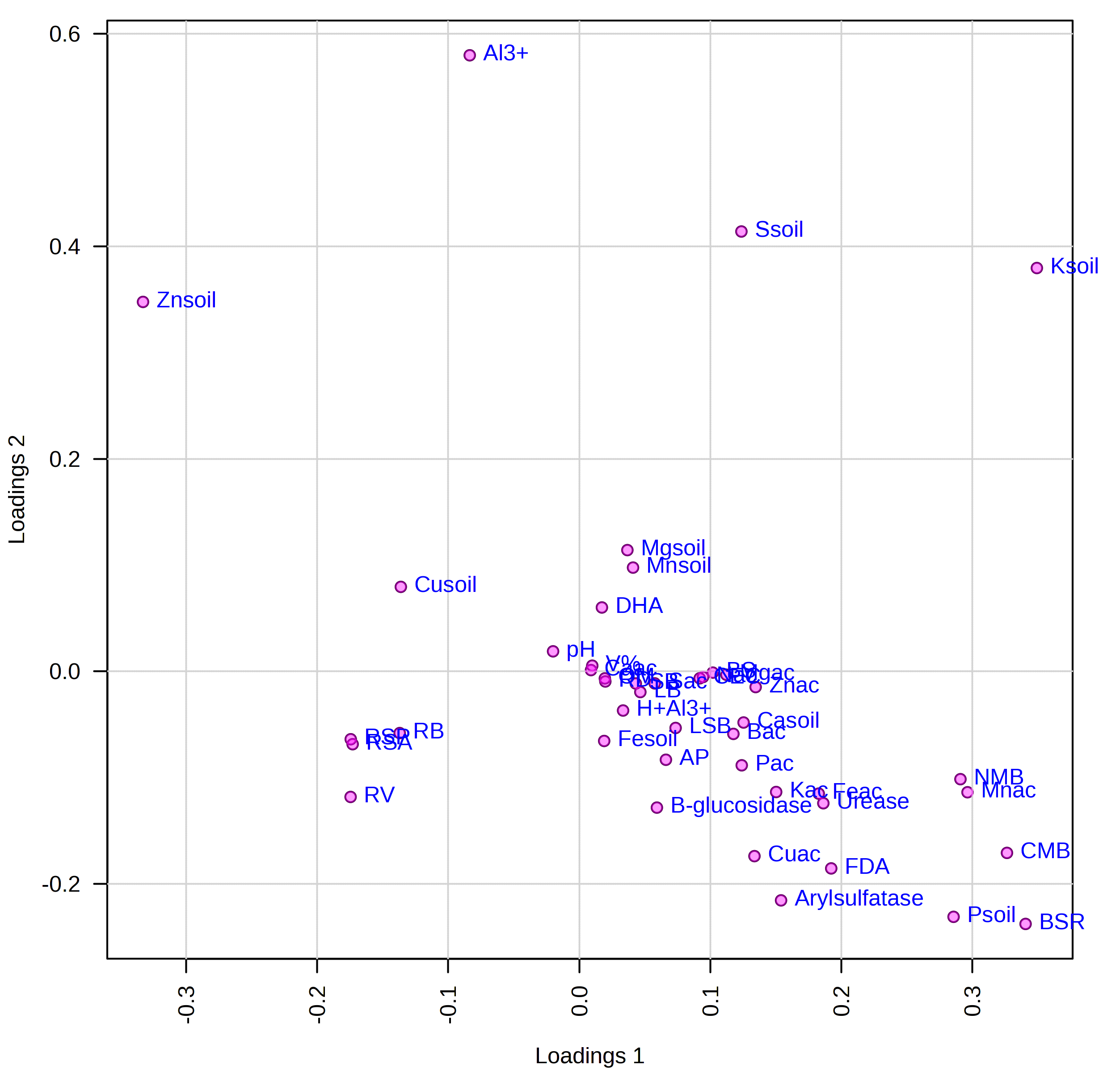
**Supplementary Figure 2.** Loading plot from Partial Least Squares Discriminant Analysis (PLS-DA), showing the contributions of soil chemical and microbiological attributes and nutrient accumulation in sugarcane to the formation of PC1 and PC2


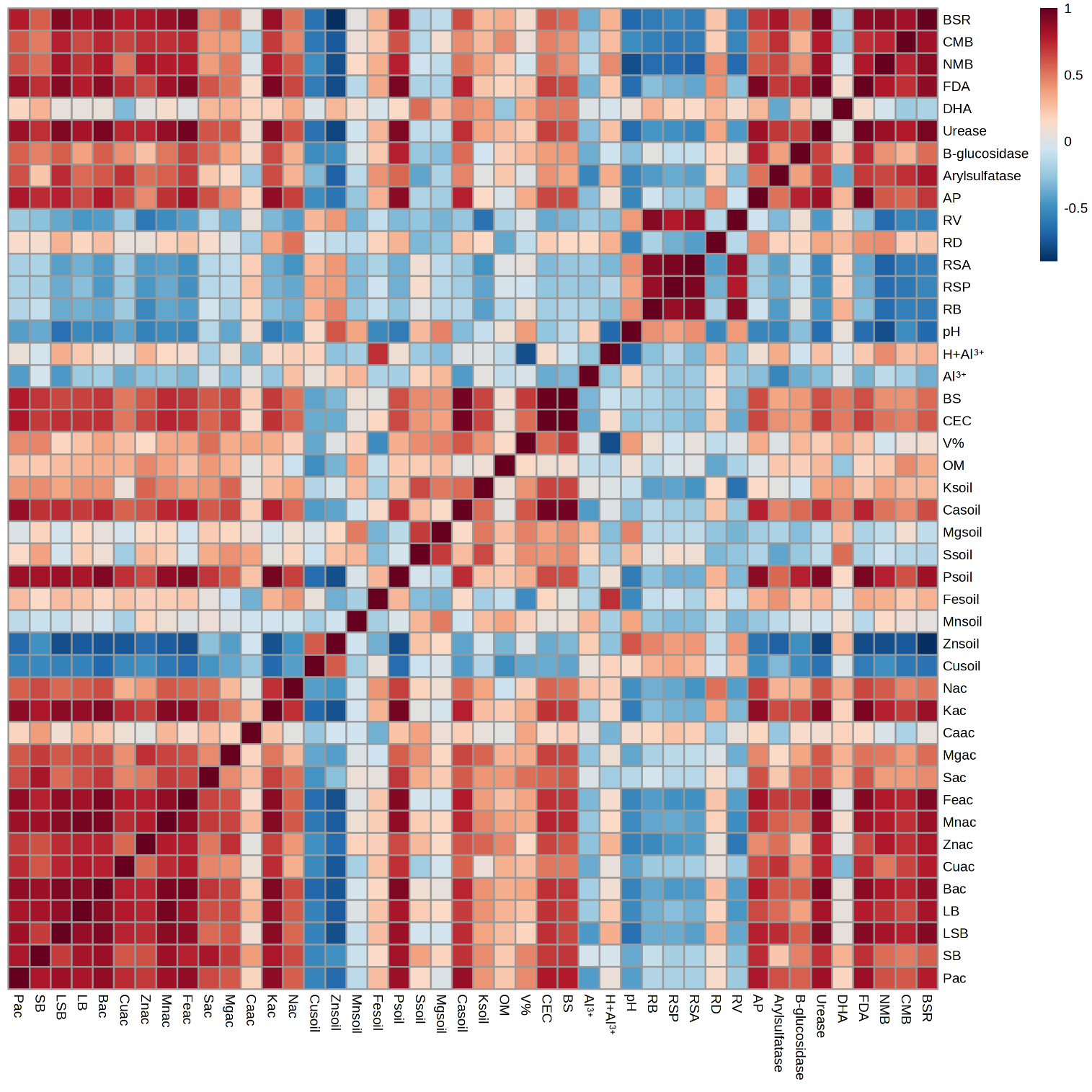
**Supplementary Figure 3.** Pearson correlation matrix among soil chemical and microbiological attributes, root traits, and nutrient accumulation in sugarcane. Positive and negative correlations are represented by shades of blue and red, respectively, with color intensity indicating the strength of the correlation (*p*<0.05)


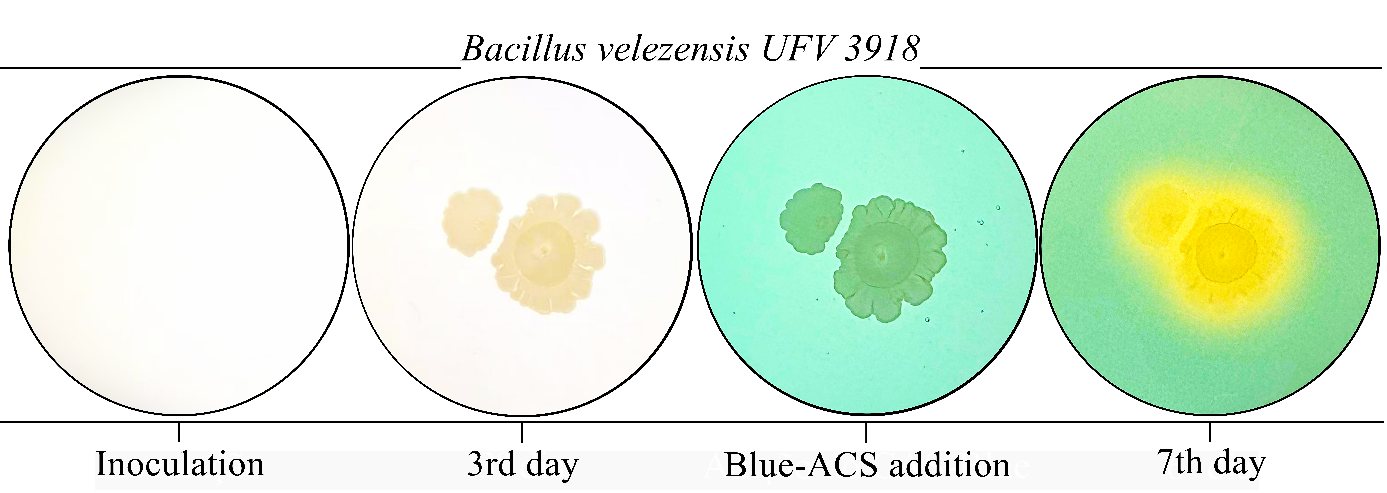


**Supplementary Figure 4.** Qualitative evaluation of siderophore production by Bacillus velezensis UFV 3918 on CAS agar medium. The formation of yellow-orange halos around the colonies indicates siderophore secretion, demonstrating the strain’s ability to chelate iron under iron-limited conditions
